# PROVGEN: A Privacy-Preserving Approach for Outcome Validation in Genomic Research

**DOI:** 10.56553/popets-2026-0064

**Published:** 2026

**Authors:** Yuzhou Jiang, Tianxi Ji, Erman Ayday

**Affiliations:** Case Western Reserve University; Texas Tech University; Case Western Reserve University

**Keywords:** Genomic privacy, differential privacy, genome-wide association studies, reproducibility

## Abstract

As genomic research has grown increasingly popular in recent years, dataset sharing has remained limited due to privacy concerns. This limitation hinders the reproducibility and validation of research outcomes, both of which are essential for identifying computational errors during the research process. In this paper, we introduce PROVGEN, a privacy-preserving method for sharing genomic datasets that facilitates reproducibility and outcome validation in genome-wide association studies (GWAS). Our approach encodes genomic data into binary space and applies a two-stage process. First, we generate a differentially private version of the dataset using an XOR-based mechanism tailored to biological characteristics. Second, we restore data utility by adjusting the Minor Allele Frequency (MAF) values in the noisy dataset to align with public MAFs using optimal transport. Finally, we convert the processed binary data back into its genomic representation and publish the resulting dataset. We evaluate PROVGEN on three real-world genomic datasets and compare it with local differential privacy and three synthesis-based methods. Our results show that PROVGEN overall outperforms existing approaches in detecting GWAS outcome errors, preserving data fidelity, and resisting membership inference attacks (MIAs). By adopting our method, genomic researchers will be inclined to share differentially private datasets while maintaining high data quality for reproducibility of their findings.

## 1 Introduction

Recent advancements in genome sequencing have unlocked significant research opportunities in genomics. Through computational and statistical methods, such as genome-wide association studies (GWAS), researchers have identified numerous associations between diseases/traits and genes, further enriching our understanding of the field.

As genomic research becomes increasingly popular, **unintentional errors** may occur during the research process or when reporting outcomes. Thus, medical professionals, who often rely on GWAS outcomes for clinical applications such as treatment procedures, need to ensure that these results are accurately computed. However, validating these results is challenging, as they typically lack access to the original datasets due to privacy concerns regarding genomic datasets [29]. As a result, the inability to validate the outcomes compromises the assessment of their quality and correctness, which in turn hampers the progress of genomic research.

This issue underscores the critical role of reproducibility in scientific research, particularly in the field of genomics. Formally, reproducibility refers to the ability to obtain consistent experiment results using the same input data, methods, and tools. Over the past decades, thanks to promotion by researchers [7, 21, 48] and the government [31], there has been a growing awareness of the importance of reproducibility, and more researchers are willing to share the datasets along with their research outcomes. However, the sharing of genomic datasets poses significant challenges due to the sensitive nature of the data involved [29]. For instance, if a genomic dataset is shared publicly, an attacker might infer with high confidence whether a particular victim has a specific trait or disease [55]. This kind of exposure represents a significant threat to personal privacy and could result in severe consequences, such as discrimination or safety risks.

Hence, there is a crucial need for an approach to validate GWAS outcomes that (i) ensures the individuals’ genomic privacy, safeguarding against state-of-the-art inference attacks, and (ii) enables validation of the research outcomes while maintaining the integrity and statistical accuracy of the data in the shared datasets. This will enable recipients (verifiers) to reproduce research outcomes and detect minor miscalculations made by researchers.

Existing works aim to enable validation of GWAS outcomes in a privacy-preserving manner. However, they either suffer from inherent ambiguity in detecting small errors [22] or provide insufficient utility to support reliable GWAS outcome validation [24, 25, 50, 51]. In this paper, we propose PROVGEN (PRivacy-preserving Outcome Validation for GENomic Data), a novel framework that securely shares genomic datasets while ensuring reproducibility in GWAS outcome validation. We focus on datasets of point mutations in DNA, namely Single Nucleotide Polymorphisms (SNPs, introduced in Section 3.1), as they are the most popular ones in biomedical research [32] and genome-wide association studies (GWAS) [10]. Note that the shared dataset is **not intended** for the primary use of data (e.g., conducting research) by medical experts, due to the noise introduced by differentially-private mechanisms. In this work, we focus on the secondary use of genomic datasets, i.e., **reproducibility and validation of GWAS outcomes** by other researchers, where noise in the shared dataset can be tolerated.

The overall procedure of the proposed scheme can be described in two stages: data perturbation and utility restoration. In the **data perturbation** stage, genomic data is initially encoded into binary values and then perturbed by XORing it with binary noise. The probability distribution used for generating the noise is carefully calibrated by leveraging the column-wise correlation of the SNPs from publicly available datasets. The core part of the data perturbation process is the **efficient binary noise generation (EBNG)** (see Definition 3), which is an improvement of the XOR mechanism initially proposed in [24]. The noise sampling of the original XOR mechanism is extremely time-consuming, making it impractical for large datasets such as genomic datasets. In contrast, our method addresses this limitation by analyzing the upper bounds of the marginal probabilities of each noise element. In the **utility restoration** stage, we introduce a post-processing scheme aiming to improve the GWAS utility distorted by the noise introduced in the first stage. We adjust the Minor Allele Frequencies (MAFs) in our dataset to align with the MAF values that are publicly available or provided along with the research findings by modifying certain values in the shared dataset. This postprocessing step significantly improves the GWAS outcomes derived from the noisy dataset, thereby enabling reliable validation of GWAS results using the final shared dataset.

We evaluate our proposed scheme using three genomic datasets from the OpenSNP project [40], focusing on four aspects: GWAS outcome validation, data fidelity, resistance to membership inference attacks (MIAs), and computational efficiency. For comparison, we implemented five alternative approaches: local differential privacy (LDP) [25], the vanilla XOR mechanism [24], GAN-AG [51], and two differentially private synthetic data generators, DPSyn [27] and PrivBayes [54]. Our results show that PROVGEN consistently outperforms all five methods in detecting subtle errors in GWAS outcomes. In contrast, the alternatives either fail to identify significant errors (e.g., LDP [25]), suffer from severe degradation in data fidelity (GAN-AG [51]), or exhibit prohibitive time complexity (vanilla XOR [24], DPSyn [27], and PrivBayes [54]). Furthermore, in terms of statistical fidelity (e.g., mean and variance errors) and robustness against MIAs, our scheme achieves superior performance in most cases. Overall, the experimental results highlight the effectiveness and practicality of PROVGEN for GWAS outcome validation in real-world genomic research settings.

Our main contributions are summarized as follows.

- We propose PROVGEN, a novel approach that enables outcome validation for GWAS via privacy-preserving genomic dataset sharing.
- We design an innovative two-stage scheme under differential privacy that effectively detects unintentional errors in GWAS outcomes.
- We develop a novel XOR-based method, inspired by Ji et al. [24], significantly reducing the time complexity of noise generation.
- We evaluate our scheme on three real genomic datasets and demonstrate that it outperforms existing methods in terms of accuracy, utility, and privacy.

## 2 Related Work

### 2.1 Reproducibility

Reproducibility ensures that experiment results are consistent in the same setting (e.g., using identical input data and methods), which facilitates research quality assessment [21] and advances scientific knowledge [28]. It has been promoted for several years by both researchers and government [7, 48]. Researchers typically share datasets used in their research such that everyone can reconstruct the same experiments and validate their results. Some examples of these datasets include ImageNet [43] and the Iris dataset [19].

However, certain datasets such as genomic and location datasets, may contain sensitive data, and thus basic anonymization techniques, e.g., hiding identifiable information, may not sufficiently protect against adversarial attacks [15]. Halimi et al. [22] propose a framework for validating GWAS results by comparing published MAFs with a noisy researcher-side MAF using a threshold derived from public genomic data. However, this method has inherent ambiguity, making the detection of small errors unreliable. Cryptographic approaches, such as homomorphic encryption (e.g.,[45]) and zero-knowledge proofs (e.g., VerDP[34]), have been explored for secure GWAS validation. However, their high computational overhead and incompatibility with current GWAS data-sharing practices limit their feasibility for large-scale genomic studies. In this paper, we address this challenge by proposing a differentially private scheme for sharing genomic datasets while preserving high data utility. Our approach enables researchers to reproduce GWAS studies conducted on the original dataset while ensuring strong privacy protection for individuals.

### 2.2 Privacy-Preserving Dataset Sharing

Differential privacy has become the standard approach for releasing aggregated statistics in a privacy-preserving manner. It introduces calibrated noise to query results to limit the amount of information that an attacker can infer from the released data. Many methods have applied differential privacy to data sharing. For example, Chanyaswad et al. [11] apply Gaussian noise in matrix form to sanitize numerical datasets, and Ji et al. [24] propose the XOR mechanism to release binary datasets using matrix-valued Bernoulli noise. Andr’es et al. [4] introduce Laplace noise for geolocation data to achieve geo-indistinguishability, a variant of differential privacy. In the genomic domain, Backes et al. [6] propose epigeno-indistinguishability to protect epigenetic microRNA data. Although these approaches have advanced the field, preserving data utility while ensuring privacy for complex biomedical and high-dimensional genomic datasets remains challenging and serves as the motivation for PROVGEN.

Meanwhile, there are also attempts to share datasets by synthesizing them under differential privacy guarantees. For example, Li et al. [27] propose a scheme that generates synthetic datasets by concerning pairwise marginal distribution of features and auxiliary information. Zhang et al. [54] synthesize datasets using Bayesian networks, where conditional probabilities are noisy and protected under differential privacy. Yelmen et al. [51] propose a GAN-based approach, which generates artificial genomic sequences by capturing internal correlations within the original dataset. However, its utility significantly degrades as the DNA sequence length increases. In this paper, we propose a privacy-preserving dataset sharing scheme for GWAS outcome validation that effectively detects subtle errors while maintaining high data utility. We compare our approach with existing methods and demonstrate its superior performance in Section 7.

### 2.3 Genomic Privacy

Encryption-based approaches [5, 8] are often inefficient concerning computational and communication costs, so the implementation of such approaches is hardly practical. Instead, differential privacy is heavily adopted. Uhler et al. [49] propose a method to release GWAS statistics (e.g., $\chi^{2}$), and Yu et al. [53] improve this work by allowing an arbitrary number of case and control individuals while considering auxiliary information. Yilmaz et al. [52] consider the correlations between SNPs and propose dependent local differential privacy to release individual genomic records. Yet, it only works for individual genomic sequences and cannot be extended to dataset sharing. Our approach publishes entire genomic datasets under differential privacy, while preserving high data utility and essential GWAS statistical properties.

## 3 Preliminaries

### 3.1 Genomic Data

DNA encodes genetic information using nucleotide bases (A,T,C,G), with SNPs representing single-base variations. A Single Nucleotide Polymorphism (SNP) is the most common type of genetic variation, where a single nucleotide differs at a specific position in the genome among individuals. For a variation to be classified as an SNP, it must occur in at least 1% of the population [2].

Each SNP can have different alleles, representing the possible nucleotide variations at a given position. The major allele is the more frequently occurring variant in a population, while the minor allele is the less common one. For example, if a specific SNP location contains C in most individuals but T in minority, then C is the major allele, and T is the minor allele. The SNP value reflects the count of minor alleles in an individual’s genome:

- **0**: homozygous for the major allele
- **1**: heterozygous, carrying one copy of the minor allele
- **2**: homozygous for the minor allele

Over 600 million SNPs have been identified across global populations, and they play a crucial role in Genome-Wide Association Studies (GWAS), where researchers investigate genetic variations linked to specific diseases or traits [33].

### 3.2 Genome-Wide Association Studies

Genome-wide association studies (GWAS) are a widely used approach for identifying correlations between genetic variations and specific traits or phenotypes [9, 16, 26, 47]. In a typical GWAS, individuals are divided into **case** and **control** groups based on the presence or absence of a particular characteristic, where the case group exhibits the trait, and the control group does not.

A **contingency table** is constructed to summarize the statistical distribution of SNP values between these groups, as shown in Table 1. In this table, $S_{i}$ represents the number of individuals in the case group with an SNP value of i at a specific genomic position, while $R_{i}$ denotes the corresponding count in the control group. The total number of individuals across both groups is denoted by N.

For example, in a study on **lactose intolerance**, if $S_{2}=10$, it means that 10 individuals with lactose intolerance are homozygous for the minor allele at that SNP position.

In a typical GWAS, researchers collect genomic data from individuals with and without a specific trait or disease, forming case and control groups. The data is preprocessed and used to construct contingency tables that summarize SNP distributions across the groups. Statistical tests such as the $\chi^{2}$ test are then applied to identify significant SNPs, and the resulting associations are published to support further research and validation.

### 3.3 Differential Privacy

Differential privacy (DP) quantifies privacy and limits the inference of any single individual from observing the query results between neighboring databases. The formal definition is as follows:

Definition 1 (**Differential Privacy**). [17] *For any neighboring datasets* $D,D^{\prime }$ *that differ only in one data record, a randomized algorithm* ℳ *satisfies* ϵ-*differential privacy if for all possible outputs* 𝒮⊆Range(ℳ)

$$\text{Pr}(ℳ(D)\in 𝒮)\leq e^{\epsilon }*\text{Pr}\left(ℳ\left(D^{\prime }\right)\in 𝒮\right).$$

The privacy parameter ϵ quantifies potential leakage: smaller values imply stronger privacy, while ϵ=∞ represents no privacy protection.

Two key properties of differential privacy ensure its robustness:

Proposition 3.1 (**Post-Processing**). [18] *If* ℳ *satisfies* ϵ-*differential privacy, then for any (possibly randomized) function* f *that does not depend on the private data, the composed mechanism* f∘ℳ *also satisfies* ϵ-*differential privacy*.

This means that any computation performed on the output of a DP mechanism without direct access to the raw data cannot weaken its privacy guarantee.

Proposition 3.2 (**Composability**). [18] *If two independent mechanisms* ${ℳ}_{1}$ *and* ${ℳ}_{2}$ *satisfy $\epsilon_{1}$* - *and* $\epsilon_{2}$-*differential privacy, respectively, then their joint release* (${ℳ}_{1}(D),{ℳ}_{2}(D)$) *satisfies* ($\epsilon_{1}+\epsilon_{2}$)-*differential privacy*.

This property enables reasoning about end-to-end privacy in multi-stage releases, such as jointly publishing a perturbed dataset and differentially private statistics.

### 3.4 The XOR Mechanism

Since we utilize an improved version of it, we also revisit the definition and privacy guarantee of the XOR mechanism proposed in [24].

Definition 2 (XOR Mechanism). *Given a binary- and matrix-valued query* $\eta_{x}$ *mapping a dataset to a binary matrix, i.e.,* $\eta_{x}:D\to \{0,1{\}}^{n\times p}$, *the XOR mechanism is defined as*

$$XOR\left(\eta_{x}(D),ℬ\right)=\eta_{x}\left(D\right)⊕ℬ,$$

*where* ⊕ *represents the XOR operator, and* ℬ, *a binary matrix noise within* $\in \{0,1{\}}^{n\times p}$, *follows a matrix-valued Bernoulli distribution with a quadratic exponential dependence structure, i.e.,*

$$ℬ\sim {\text{Ber}}_{n,p}\left(\Theta ,{𝚲}_{1,2},\cdots ,{𝚲}_{n-1,n}\right).$$

#### 3.4.1 PDF of Matrix-valued Bernoulli Distribution.

The PDF of this matrix-valued Bernoulli distribution with quadratic exponential dependency, i.e., $ℬ\sim {\text{Ber}}_{n,p}\left(𝚯,{𝚲}_{1,2},\cdots ,{𝚲}_{n-1,n}\right)$ is parameterized by matrices $𝚯,{𝚲}_{1,2},\cdots ,{𝚲}_{n-1,n}\in {ℛ}^{p\times p}$ and is expressed as

(1) $$f_{ℬ}\left(B\right)=C\left(𝚯,{𝚲}_{1,2},\cdots ,{𝚲}_{n-1,n}\right)\times \text{exp}\left\{\text{Tr}\left[B𝚯B^{T}\right]+\sum_{i=1}^{n}\sum_{j\neq i}^{n}\text{Tr}\left[J_{ij}B{𝚲}_{i,j}B^{T}\right]\right\},$$

where

$$C\left(𝚯,{𝚲}_{1,2},\cdots ,{𝚲}_{n-1,n}\right)={\left[\sum_{B_{k}}\text{exp}\left\{\text{Tr}\left[B_{k}𝚯B_{k}^{T}\right]+\sum_{i=1}^{n}\sum_{j\neq i}^{n}\text{Tr}\left[J_{ij}B_{k}{𝚲}_{i,j}B_{k}^{T}\right]\right\}\right]}^{-1},$$

is the normalization constant, $B_{k}\in \{0,1{\}}^{n\times p}$, and $J_{ij}$ is the matrix of order n×n with 1 at the (i,j)-th position and 0 elsewhere.

Similar to the classical differential privacy output perturbation mechanisms (such as Gaussian or Laplace mechanisms), which attain privacy guarantees by constraining the parameters of the considered distributions (i.e., Gaussian or Laplace distribution), the XOR mechanism also ensures privacy by controlling the parameters in the distribution in (1). The sufficient condition for the XOR mechanism to achieve ϵ-differential privacy is recalled as follows.

Theorem 3.3. *The XOR mechanism achieves* ϵ-*differential privacy of a matrix-valued binary query if* Θ *and* $\Lambda_{i,j}$ *satisfy*

(2) $$s_{f}(\|\lambda (\Theta ){\|}_{2}+\sum_{i=1}^{N-1}\sum_{j=i+1}^{N}{\left\|\lambda (\Lambda_{i,j})\right\|}_{2})\leq \epsilon ,$$

*where* $s_{f}$ *is the sensitivity of the binary- and matrix-valued query, and* $\|\lambda (\Theta ){\|}_{2}$ *and* ${\left\|\lambda \left(\Lambda_{i,j}\right)\right\|}_{2}$ *are the* $l_{2}$ *norm of the vectors composed of eigenvalues of* 𝚯 and ${𝚲}_{i,j}$, *respectively*.

In Section 6.1, we will discuss how to obtain $s_{f}$ when considering a binarized genomic dataset.

In practice, it is computationally prohibitive to evaluate the normalization constant (see (1)) in the PDF of the matrix-valued Bernoulli distribution; thus, to generate a sample from it, [24] resorts to the Exact Hamiltonian Monte Carlo scheme. However, this scheme is impractical for our study due to its extreme high time complexity caused by thousands of SNPs in the dataset. To address this issue, we propose a new noise generation scheme without compromising the privacy of the original XOR mechanism. In particular, each element in the noise matrix is generated using its calibrated marginal distribution. More details are deferred to Section 6.2.

## 4 System Settings

In this section, we introduce the system model and the threat model.

### 4.1 System Model

In our system, we consider a scenario in which researchers are conducting genome-wide association studies (GWAS). In this setting, we consider two key parties: **the researcher** and **the verifier**. The researcher performs GWAS on a local genomic dataset and publishes the research findings publicly. Typically, GWAS research findings include the name and methodology of the experiment, the number of samples in the case group, details about the control group (which may include aggregated statistics of the control group), the SNP identifier (commonly referred to as the **rsID**), and the minor allele frequency (MAF) of SNPs that are significantly associated with the trait or disease of interest.

To enhance the credibility of its research and reputation in the research community, the researcher would share the entire research dataset along with the findings for external validation. However, direct sharing of genomic datasets raises privacy concerns [29]. Instead, the researcher sanitizes the dataset using a privacy-preserving scheme before sharing it.

Throughout the research process, the researcher may unintentionally introduce errors, including errors during: (i) data cleaning and preprocessing, (ii) statistical analysis, or (iii) publication of findings. Our work focuses on detecting unintentional errors that occur during these stages, but excludes errors introduced during data collection. Errors at this stage are generally considered hard to detect and have not been effectively addressed by existing methods. This is widely acknowledged in the field, and no current work has provided a robust solution for identifying or correcting such errors.

Importantly, multiple errors may arise at the same or different stages. Since such errors are generally independent, we treat them as independent events. Our approach is capable of detecting such compounded errors because they collectively lead to discrepancies in GWAS results, making it easier for our scheme to identify them.

The verifier, assumed to be a peer reviewer or another researcher, seeks to validate the published findings. Using the shared dataset, the verification process proceeds as follows. First, the verifier reproduces the same GWAS experiments reported by the researcher using the shared dataset and obtains the p-values for all SNPs claimed as significant in the research findings. Then, the verifier calculates the percentage of SNPs reported as significant in the original findings that remain statistically significant in the reproduced results (possibly using a relaxed p-value threshold instead of the original p-value), which is termed the **SNP retention rate**.

To assess the trustworthiness of the findings, the verifier compares the SNP retention rate from the reproduced results to a theoretical ideal or expected rate, which represents the anticipated retention rate under error-free conditions. The primary goal of this paper is to ensure that meaningful differences in retention rates can be observed, enabling the verifier to detect potential errors. Note that the estimation of such an expected rate, which can be obtained by using additional auxiliary datasets, is beyond the scope of this paper. If this difference falls within a specified threshold, the findings are deemed reliable. Otherwise, the verifier may initiate further investigation or request additional detailed information, pending Institutional Review Board (IRB) approval.

#### 4.1.1 Motivation for Sharing Raw Genomic Data.

Publishing datasets provides several important advantages over releasing only aggregated GWAS statistics:

- **Independent validation and reproducibility**. Verifiers can locally re-run GWAS using the shared dataset and compare their results with the published statistics. This enables transparent verification of analysis steps and improves the reproducibility of reported findings.
- **Support for advanced applications**. Access to individual-level genomic data allows downstream analyses such as variant calling (identifying genetic variants relative to a reference genome) and machine learning tasks (e.g., phenotype classification). These applications are not feasible with aggregated summary statistics. Our scheme enables such use cases by sharing privacy-preserving datasets that retain high utility (see Section 7.5 and Table 4).
- **Community and benchmarking benefits**. Programs such as NIH’s TOPMed [46] and All of Us [39] repeatedly emphasize the need for shareable, individual-level simulated datasets to support reproducibility and benchmarking of new methods.

As shown in Section 7.5, our scheme achieves high data fidelity while maintaining privacy protection. This balance demonstrates that our approach can effectively support the above goals under practical privacy budgets.

### 4.2 Threat Model

In our framework, we assume that the researcher is honest yet cautious, holding the original genomic dataset without sharing it directly. Meanwhile, an honest researcher may still unintentionally provide incorrect GWAS outcomes due to computational errors, which could mislead other researchers. Our scheme aims to address this issue by offering a means to reproduce and validate GWAS experiments, enhancing their reliability.

Note that our scope does not extend to scenarios involving a malicious researcher who might intentionally fabricate datasets to report false results. Deliberately creating and using synthetic datasets to produce inaccurate findings poses a challenge that is nearly impossible in most data analysis contexts, not only in GWAS. In this case, only those with direct access to the original data can validate the authenticity of research finding. Moreover, the ethical implications and potential consequence of using fabricated dataset (e.g., damage to the researcher’s credibility and reputation from funding agencies) serve as strong deterrents against such misconduct.

The verifier may be malicious and curious about the original dataset. Such a malicious verifier, acting as an attacker, may conduct membership inference attacks (MIAs) to determine if an individual (the victim) is part of the shared dataset or not. Since individuals in a genomic dataset often share an attribute (e.g., trait or disease), linking the victim to the dataset could also associate them with that attribute. For instance, assume the researcher shares a dataset consisting of heart disease patients. An attacker could use the published research findings and the shared dataset to predict the target’s presence in the dataset. If the analysis indicates that the target is likely a member, the attacker could infer a potential association of the individual with heart-related diseases.

We assume that the attacker has access to two key pieces of information: (i) the shared dataset from the researcher and (ii) the specific trait/disease of the individuals in the dataset, e.g., heart disease in the previous example. In addition, the attacker can exploit auxiliary knowledge to launch MIAs by constructing a reference dataset of individuals without the trait/disease (e.g., from the 1000 Genomes project [3]). We consider the following MIAs: 1) Hamming distance-based test (HDT) [22], 2) decision tree, 3) random forest, 4) XGBoost [13], 6) Support Vector Machine [14], and 7) neural network. More details will be deferred to Section 7.2.3.

## 5 PROVGEN Workflow

**Figure 1:**
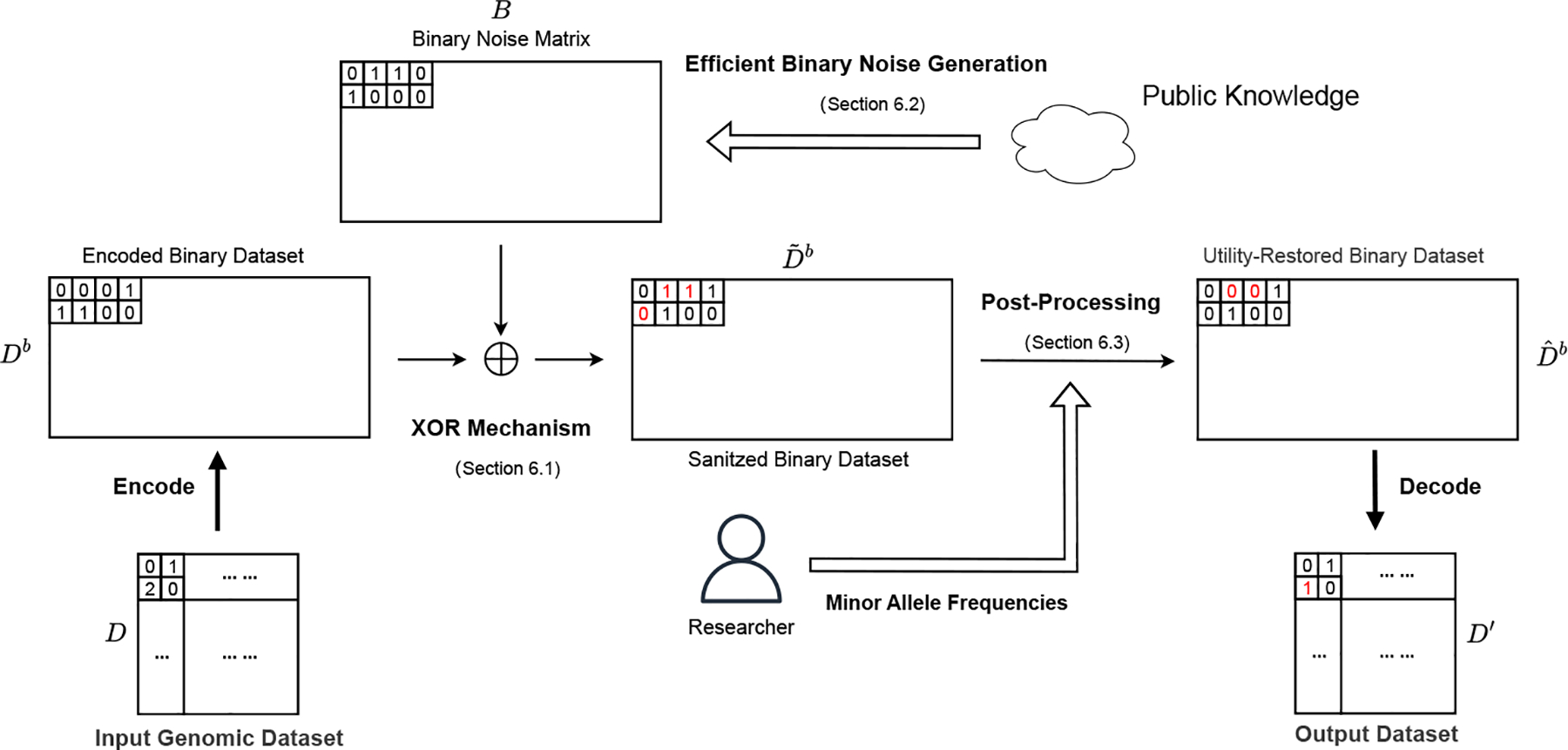
The workflow of PROVGEN operates as follows: 1) The input dataset D is encoded into a binary form $D^{b}$ and subjected to an XOR operation with binary noise, generated through Efficient Binary Noise Generation (EBNG). 2) We utilize the Minor Allele Frequencies (MAFs) of SNPs to enhance the data utility of the noisy dataset ${\overset{ˆ}{D}}^{b}$ using optimal transport. Finally, we convert the optimized binary dataset ${\overset{ˆ}{D}}^{b}$ back into its original SNP format to obtain the final shared dataset $D^{\prime }$.

Our proposed workflow is depicted in Figure 1. During the data perturbation stage, we first encode the genomic dataset into a binary matrix. Each SNP is represented using two bits while considering the biological property (discussed in Section 6.1). We then implement a noise sampling scheme, an adaptation of the XOR mechanism [24], optimized for efficient generation of large datasets. This scheme generates a noisy version of the binary matrix while considering inherent correlations among SNPs from publicly available datasets.

In the utility restoration stage, we address the utility degradation caused by noise addition. We develop a post-processing technique focused on enhancing the GWAS utility distorted in the first stage.

This involves aligning the Minor Allele Frequencies (MAFs) in the noisy dataset with those that are publicly available or have been published as a part of research findings by flipping allele values using optimal transport [1]. Following this, we convert the altered dataset back into genomic space and make it available to verifiers for validation.

## 6 Methodology

### 6.1 Genomic Dataset Perturbation

Existing methods are not effective for genomic data due to two reasons: they either exhibit high time complexity [27, 54] or fail to appropriately address the inherent correlation among SNPs [25], leading to significant utility loss, as evidenced by our preliminary experiments. We overcome these challenges by converting genomic datasets into binary space. Specifically, we encode each SNP value to 2 bits according to the conversion metric shown in Table 2 and generate a binary version of the genomic dataset $D^{b}\in \{0,1{\}}^{n\times 2m}$. It is important to note that this binary representation of SNPs is consistent with their biological characteristics (refer to Section 3.1). As detailed in Section 3.2, SNP data have three values (0, 1 and 2) that indicate the number of minor alleles in a gene. Each allele, inherited from one parent, contributes to the SNP value: ‘00’ for value 0 (no minor allele), ‘01’ for value 1 (one minor allele), and ‘11’ for value 2 (both parents with a minor allele). The binary matrix resulting from this encoding effectively simulates the allele distribution in the genomic sequence. Therefore, flipping one binary value in the binary dataset is analogous to flipping one allele, thus maintaining biological consistency in our data representation.

After encoding, we implement the XOR mechanism [24] to perturb the binary-encoded SNP dataset. The perturbation is represented as ${\tilde{D}}^{b}=D^{b}⊕B$, where $D^{b}$ is the original binary SNP dataset, and ${\tilde{D}}^{b}$ is the perturbed outcome. The operator ⊕ denotes the (exclusive or) XOR operation, and $B\in \{0,1{\}}^{n\times 2m}$ is the binary noise matrix, sampled from the matrix-valued Bernoulli distribution.

The parameters of this distribution are calibrated with respect to the privacy parameter ϵ and the sensitivity $s_{f}$ of the binary encoding between D and $\overset{ˆ}{D}$, where $D\sim \overset{ˆ}{D}$ denotes neighboring genomic datasets that differ by a single individual’s genomic record. Mathematically, the sensitivity is defined as:

(3) $$s_{f}=\underset{D,\overset{ˆ}{D}}{\text{sup}}\;{\left\|D^{b}⊕{\overset{ˆ}{D}}^{b}\right\|}_{F}^{2}.$$

Here, $\|\cdot {\|}_{F}^{2}$ denotes the squared Frobenius norm, which, in the case of binary matrices, equals the number of differing entries (i.e., the Hamming distance). For example, if the dataset contains 1,000 SNPs and each SNP is encoded using 2 bits, then a single individual’s record contributes up to 2 × 1,000 = 2,000 bits, which defines the worst-case sensitivity.

#### Motivation of Adopting XOR Mechanism.

Our approach is motivated by the need to preserve two fundamental types of correlations inherent in genomic data: (i) SNP-wise correlations, which capture dependencies between SNPs, and (ii) sample-wise correlations, which reflect kinship relationship among individuals, such as those observed in family members. Although local differential privacy (LDP) [25] can be employed within our framework as an alternative for the XOR mechanism and achieves comparable utility for GWAS reproducibility, it does not account for these correlations. As demonstrated by Yilmaz et al. [52], neglecting such dependencies during differentially private perturbation can expose the shared dataset to powerful inference attacks. To address this vulnerability, we adopt the XOR mechanism, which generates binary noise matrices sampled from a matrix-valued Bernoulli distribution with a quadratic exponential dependency structure [30]. This formulation naturally models both SNP-wise and sample-wise dependencies, enabling correlation-aware perturbation that enhances privacy while preserving utility. As a result, the XOR mechanism is well-suited for our setting, as it offers stronger protection against inference attacks by preserving critical correlations, and it lays a robust foundation for the downstream post-processing.

It is noteworthy that, in practice, there are infinite ways to generate the parametric matrices in the XOR mechanism. As long as the sufficient condition in (2) is satisfied, achieving ϵ-differential privacy is possible. In [24], the authors assume that these matrices are positive definite and propose generating them via a computationally intensive optimization procedure. In contrast, our approach relaxes this requirement by constructing the parametric matrices using biologically informed characteristics of SNPs and sample-level relationships among individuals, thereby reducing computational complexity while preserving the structural dependencies essential for both privacy and utility.

In particular, the entries of 𝚯 (cf. (1) in Section 3.4.1) are referred to as “feature-association” values [24, 30]. They model the correlations between the columns of $D^{b}\in \{0,1{\}}^{n\times 2m}$, representing the inherent dependencies among SNPs [36]. To preserve these inherent correlations, we utilize a publicly available reference dataset that contains the same set of SNPs as D (e.g., the control group in a casecontrol study). Such datasets can be obtained from open-source genomic repositories, such as the 1000 Genomes Project [3].

To model these correlations, we employ a **log-linear association approach**, constructing a matrix $\tilde{𝚯}\in R^{2m\times 2m}$ with diagonal entries ${\tilde{\theta }}_{p,p}$ and non-diagonal entries ${\tilde{\theta }}_{p,q}(p\neq q)$, computed as:

(4) $${\tilde{\theta }}_{p,p}=\text{log}\frac{\text{Pr}\left(M_{p}=0\right)}{\text{Pr}\left(M_{p}=1\right)},\;p\in \left[1,2m\right],\;{\tilde{\theta }}_{p,q}=\text{log}\frac{\text{Pr}\left(M_{p}=0,M_{q}=1\right)\;\text{Pr}\left(M_{p}=1,M_{q}=0\right)}{\text{Pr}\left(M_{p}=1,M_{q}=1\right)\;\text{Pr}\left(M_{p}=0,M_{q}=0\right)},$$

where p,q∈[1,2m],p≠q,M is the binarized version of a publicly available dataset, $\text{Pr}\left(M_{p}=0\right)$ represents the frequency of values equal to 0 in the p-th column, and $\text{Pr}\left(M_{p}=0,M_{q}=1\right)$ denotes the frequency of tuples where the value is 0 in the p-th column and 1 in the q-th column.

The entries of ${𝚲}_{ij}$, referred to as “object-association” values, model the correlations between rows i and j of $D^{b}$, representing kinship relationships [23]. If the genomic dataset contains SNP sequences of family members, ${𝚲}_{ij}$ can be derived using Mendel’s law. Given the privacy parameter ϵ, we determine the distribution parameters (𝚯 and ${𝚲}_{ij}$) based on correlations obtained from publicly available genomic datasets of the same nature. In this scenario, ${𝚲}_{ij}$ is generated using the log-linear association approach to preserve kinship-based correlations.

By invoking Theorem 3.3, we have

(5) $$s_{f}(\|\lambda (𝚯){\|}_{2}+\sum_{i=1}^{n-1}\sum_{j=i+1}^{n}{\left\|\lambda ({𝚲}_{i,j})\right\|}_{2})\leq \epsilon_{x},$$

as a sufficient condition to protect the privacy of genomic dataset without kinship correlations.

### 6.2 Efficient Binary Noise Generation

The original noise generation of the XOR mechanism [24] is time consuming as it constructs the entire binary noise matrix $B\in \{0,1{\}}^{n\times 2m}$ at once by repeatedly simulating Hamiltonian dynamics until the system reaches convergence. Such a procedure involves repeatedly computing energy gradients (i.e., derivatives of the system’s energy function) and updating momentum variables over many iterations, which results in substantial computational overhead. Thus the noise generation procedure in [24] is impractical for large size genomic datasets where both n (the number of samples) and m (the feature dimension) can be extremely large.

In this section, we introduce a novel sampling scheme, termed **efficient binary noise generation** (EBNG). The proposed scheme accelerates the noise generation process, making it practical for large-scale genomic datasets. The key idea is to bypass the Hamiltonian Monte Carlo sampling procedure used in [24] and instead generate the entries of B in an element-wise manner. To make this alternative solution feasible, we need to calibrate every marginal probability of $\text{Pr}\left[B_{u}=1\right]$ so that (i) the target correlation structure 𝚯 across entries is preserved and (ii) the original privacy guarantee in Theorem 3.3 still holds.

First, we review the following lemma which connects the matrix-valued Bernoulli distribution with the multivariate Bernoulli distribution. By leveraging the connection between two distributions, we can first generate the noise in a vector formate, i.e., $\text{vec}\left(B^{T}\right)$, then respahe it as a matrix.

Lemma 6.1. [30] *If* $ℬ\sim {\text{Ber}}_{n,p}\left(𝚯,{𝚲}_{1,2},\cdots ,{𝚲}_{n-1,n}\right)$, *then* $b=\text{vec}\left({ℬ}^{T}\right)\in \{0,1{\}}^{np\times 1}$ is *attributed to a multivariate Bernoulli distribution with parameter* 𝚷, *i.e*., $\text{vec}\left({ℬ}^{T}\right)\sim {\text{Ber}}_{np}(𝚷)$, *and*

$$f_{\text{vec}\left({ℬ}^{T}\right)}\left(\text{vec}\left({ℬ}^{T}\right)=b\right)=C(𝚷)\text{exp}\left\{b^{T}𝚷b\right\}.$$

*The parameter* 𝚷 *and the normalization constant* C(𝚷) *are*

(6) $$𝚷=I_{n}⊗𝚯+\sum_{i=1}^{n}\sum_{j\neq i}^{n}J_{ij}⊗{𝚲}_{i,j},\;\text{and}\;C(𝚷)={\left[\sum_{b\in 𝒮}\text{exp}\left\{b^{T}𝚷b\right\}\right]}^{-1},\;𝒮={\left\{0,1\right\}}^{np\times 1},$$

where ⊗ is the Kronecker tensor product.

As will be discussed in Section 7, the considered SNP datasets do not have kinship correlations, thus we can adopt a simple form of the multivariate Bernoulli distribution, i.e.,

(7) $$f_{b}\left(b=b\right)=C\left(𝚷\right)\text{exp}\left\{b^{T}𝚷b\right\},\;𝚷=I_{n}⊗𝚯.$$

However, the normalization constant in (7), i.e., C(𝚷), involves a summation of $2^{nm}$ values and is still intractable. Thus, we will generate each element of the noise vector separately, based on an approximated marginal PDF of each element, $B_{u}$. In what follows, we first present the efficient noise generation scheme, then derive the approximated marginal PDF of $B_{u}$ and provide with the privacy guarantee. The detailed proof is deferred to Appendix D.

Definition 3 (Efficient Binary Noise Generation). *Given a genomic dataset D, suppose that each individual has m SNPs (i.e.,* P=2m *SNP bits after encoding). The efficient binary noise generation scheme perturbs the* u-*th bit* (u∈[1,2m]) *for each individual with a random binary bit* $B_{u}$, *and* $\text{Pr}\left[B_{u}=1\right]$ *is calibrated based on the correlation among the SNP bits*.

A sufficient condition for the above bit-wise perturbation to preserve ϵ-differential privacy on the entire genomic dataset D is shown below.

Theorem 6.2. *Let* 𝚷 *be the parameter determined in* (7), $\text{SUM}\left({𝚷}_{u}\right)$ *is the summation of the uth row of* 𝚷, *and* ${𝚷}_{u,u}$ *is the uth diagonal entry of* 𝚷. *Define*

$$\kappa_{u}=2\times \text{SUM}\left({𝚷}_{u}\right)-{𝚷}_{u,u},$$

*then, Definition 3 achieves* ϵ-*differential privacy if we use the following approximated marginal PDF for each noise bit $B_{u}$*

(8) $$\text{Pr}\left[B_{u}=1\right]=\left\{\begin{array}{ll} \frac{1}{2} & \text{if}\;\kappa_{u}>\|\lambda (𝚯){\|}_{2}\\ \frac{1}{1+\text{exp}\left(\kappa_{u}\right)} & \text{if}\;\kappa_{u}\leq \|\lambda (𝚯){\|}_{2} \end{array}\right.,$$

*where* $\|\lambda (𝚯){\|}_{2}$ *is the* $l_{2}$ *norm of the eigenvalues of* 𝚯.

Our new binary noise sampling scheme relies only on simple algebraic operations and a sequence of Bernoulli draws, so it is substantially more efficient than methods that require repeated Hamiltonian dynamics simulation. Table 3 summarizes the key differences between our noise generation approach and that of Ji et al. [24]. As further demonstrated in Section 7.7, the original XOR mechanism can generate only about 10 SNPs within a one-hour runtime, confirming that the vanilla method is impractical for our setting.

### 6.3 Restoring GWAS Utility via Post-Processing

Efficient binary noise generation introduces an excessive amount of noise into the encoded genomic dataset. This poses a significant challenge for verifiers, as the resultant dataset from the XOR mechanism compromises reliable GWAS outcome validation due to substantial utility loss. To address this issue, we employ a post-processing strategy that leverages public Minor Allele Frequencies (MAFs) (or shared by the researcher as a part of the research findings) to enhance the dataset’s utility. Notably, sharing of MAF values is permitted under the NIH Genomic Data Sharing (GDS) policy [35] and is commonly included as a part of GWAS research findings.

In our approach, the published MAFs are represented as

(9) $${ℳ}^{r}=\left\{{ℳ}_{0}^{r},{ℳ}_{1}^{r},\ldots ,{ℳ}_{m}^{r}\right\},$$

where ${ℳ}_{j}^{r}$ denotes the MAF of the j-th SNP in the target dataset. We also compute the MAFs, denoted as $\tilde{ℳ}$, from the dataset generated in the first stage.

Our objective is to align the MAF distribution of the noisy dataset ${\tilde{D}}^{b}$ with the published MAFs while mitigating utility degradation, namely, minimizing the number of allele flips required. The postprocessing procedure for each SNP j follows these steps:

1. Compute the MAF value ${\tilde{ℳ}}_{j}$ of the SNPs in the noisy (binarized) dataset ${\tilde{D}}^{b}$.
2. Apply an optimal transport approach, specifically the earth mover’s distance [42], to transition from ${\tilde{ℳ}}_{j}$ to the reference MAF ${ℳ}_{j}^{r}$. This approach determines the percentage of alleles that need to be flipped and their corresponding values.
3. Determine the exact number of alleles to be flipped by applying the floor function to the product of the computed percentage and the total number of alleles.
4. Randomly select the determined number of alleles and flip them.

Further details regarding this optimization problem can be found in Appendix E.

After post-processing, we convert the dataset back into genomic space following Table 2. During this step, any invalid binary output (e.g., “**10**”) is converted to “**1**” in SNP representation. This adjustment aligns with biological properties and enhances data utility while maintaining the integrity of the original genomic information.

#### End-to-end Privacy Analysis.

Our scheme does not provide a formal mathematical privacy guarantee for the joint release of the shared dataset and the accompanying Minor Allele Frequencies (MAFs). This is because MAF statistics are released without obfuscation under the NIH Genomic Data Sharing (GDS) policy [35], which treats them as public information (i.e., ϵ=∞). Consequently, the formal ϵ-differential privacy guarantee applies only to the perturbed dataset generated by the XOR mechanism. As shown in Section 7.6, our empirical evaluation against multiple membership inference attacks indicates that this configuration does not incur additional privacy leakage.

To further examine a stricter privacy configuration, we consider an alternative setting (Section 7.4.3) where MAFs are not publicly available and are instead protected under differential privacy. The shared dataset is released with privacy budget $\epsilon_{e}$, and the MAFs are released with privacy budget $\epsilon_{m}$ under the Laplace mechanism. According to the composability property of differential privacy [17], their joint release satisfies ($\epsilon_{e}+\epsilon_{m}$)-differential privacy. Experimental results show that PROVGEN continues to detect GWAS errors effectively under this configuration, demonstrating robustness even when both components are protected by differential privacy.

## 7 Evaluation

We conducted a comprehensive evaluation of our scheme using three real-world genomic datasets from the OpenSNP project [40]. For comparison, we introduced a local differential privacy approach based on randomized response [25] as a baseline. Additionally, we evaluated the state-of-the-art synthesis-based approach [51] for genomic dataset sharing to highlight its limitations on realistic genomic datasets.

Furthermore, we implemented two widely used synthetic data generation approaches, DPSyn [27] and PrivBayes [54], both of which were winners of the *NIST Differential Privacy Synthetic Data Challenge* [37]. These methods are commonly used for tabular data and serve as alternative solutions when GAN based approaches fail for genomic dataset sharing. However, as discussed, these synthesis based methods suffer from significant computational complexity and can only handle genomic datasets with approximately 130 SNPs. This is far below the millions of SNPs typically present in an individual’s genome.

### 7.1 Datasets

We leveraged the OpenSNP project [40] to construct sample datasets for evaluation. We select three phenotypes, i.e., lactose intolerance, hair color, and eye color. The corresponding datasets contain 9,091 SNPs and 60 individuals (lactose intolerance), 9,686 SNPs and 60 individuals (hair color), and 28,396 SNPs and 401 individuals (eye color). For each phenotype, we also constructed a reference dataset with matching SNPs to ensure alignment with the target data.

### 7.2 Evaluation Metrics

In this section, we introduce evaluation metrics regarding GWAS outcome validation, data fidelity, resistance against MIAs, and time complexity.

As introduced in Section 4.1, we use the SNP retention rate to assess the reliability of GWAS findings. This rate quantifies the proportion of significant SNPs from the original study that remain significant when reproduced using the shared dataset. Since we do not prescribe a specific threshold for acceptability, our evaluation focuses on the difference in SNP retention rates between error-free and error-injected scenarios. A difference greater than zero that shows clear separation across the x-axis (e.g., error rates) indicates the presence of potential errors. Larger deviations correspond to higher confidence in error detection. To explore how such deviations arise, we model two representative types of errors commonly encountered in GWAS pipelines.

#### 7.2.1 GWAS Outcome Validation.

In an honest research setting, a researcher may inadvertently report inaccurate SNPs as part of GWAS outcomes. We categorize such errors into two types and model them accordingly. Unintentional errors may occur in many other ways as well, but we use the below simple scenarios to illustrate the consequences of such errors.

**Flipping error** occurs when some p-values are calculated incorrectly. This type of error may arise from mistakes during any of the five stages mentioned in Section 3.2: data collection, group formation, preprocessing, statistical analysis, or publication of findings. We model flipping errors by randomly selecting a portion of the p-values and replacing them with values between 0 and 1. The error rate, denoted by $\delta_{f}$, parameterizes this error. The researcher correctly reports $1-\delta_{f}$ of the truly significant SNPs, while the remaining $\delta_{f}$ are reported incorrectly.

**Noise error** happens during all stages except for the publication of findings. This type of error introduces noise into contingency tables or the calculated p-values. We model noise errors by adding normally distributed noise to each p-value reported in the research findings. The error rate $\delta_{n}$ denotes the scale (standard deviation) of the normal distribution from which the noise is sampled.

For GWAS, we evaluate significance using two common statistical tests: the $\chi^{2}$ test and the odds ratio test. The $\chi^{2}$ test measures whether the observed distribution of SNPs significantly deviates from what is expected under no association. The odds ratio test estimates how strongly a specific SNP is associated with the trait by comparing occurrence odds between case and control groups. Further technical details of these tests are provided in Appendix C.

#### 7.2.2 Data Fidelity.

In addition to our primary objective, we assessed the performance of the proposed scheme using data fidelity metrics relevant to general-purpose data analysis. We employed metrics including average point error, average sample error, mean error, and variance error. These metrics quantify the extent to which the integrity and statistical properties of the original dataset are preserved when shared with other researchers. The following sections detail each metric.

##### Average Point Error.

Average point error measures the entry-level discrepancy between two datasets. Given two datasets, D and $D^{*}$, with dimensions n×m, we compute the number of mismatched entries and define the point error as:

(10) $${\text{Error}}_{p}=\frac{\sum_{i=1}^{n}\sum_{j=1}^{m}1_{D_{ij}\neq D_{ij}^{*}}}{n\times m}.$$

##### Average Sample Error.

Average sample error quantifies the distance between corresponding samples in two datasets. We calculate this using the $l_{1}$ norm, which represents the sum of absolute differences between each sample pair, normalized by the number of SNPs in the datasets:

(11) $${\text{Error}}_{s}=\frac{\sum_{i=1}^{n}\sum_{j=1}^{m}\left|D_{ij}-D_{ij}^{*}\right|}{n\times m}.$$

##### Mean and Variance Error.

In addition to the previous metrics, we assess mean and variance errors, which serve as key indicators of statistical data fidelity. These metrics are computed as the absolute differences in the mean and variance values within the SNP domain.

#### 7.2.3 Resistance Against Membership Inference Attacks (MIAs).

We conducted a privacy evaluation against membership inference attacks (MIAs) following existing work on genomic data [22]. We adhered to the threat model in Section 4.2 and employed multiple attacks, including the Hamming distance test and multiple machine learning-based MIAs. We do not include likelihood ratio tests in our evaluation, as done in [22], because they do not involve dataset sharing [44]. We use accuracy as our evaluation metric. For each machine learning-based MIA, we train a separate model using a balanced sample of individuals from both the shared and a reference dataset. Accuracy is then evaluated using the remaining individuals in the original, unperturbed version of the shared dataset. The details of the attack strategies are outlined below.

##### Hamming Distance Test (HDT) [22].

The Hamming distance test (HDT) is considered one of the most powerful MIAs against genomic datasets [22]. It leverages the pairwise Hamming distance between genomic sequences in the case group (received from the researcher and subjected to perturbation for privacy guarantees) and in the control group (constructed from publicly available datasets). Specifically, for each individual i in the shared dataset (the case group), a malicious client calculates the Hamming distances between individual i and all individuals in the reference dataset (the control group), then records the minimum Hamming distance for individual i. The malicious client collects all these minimum Hamming distance for individuals in the case group and selects a threshold γ following 5% false positive rate. When attempting to identify a victim, the malicious client calculates the minimum Hamming distance between the victim’s sequence and all individuals in the control group. If the minimum Hamming distance is lower than the threshold γ, the target is considered a member of the case group, and vice versa.

##### Machine Learning-Based MIAs.

We assume that the attacker employs the following machine learning-based membership inference attacks (MIAs) for inference: decision tree (DT), random forest (RF) [41], XGBoost [13], support vector machine (SVM) [38], and neural networks. Upsampling methods, such as SMOTE [12], are not suitable in this case due to the limited sample size (e.g., only 60 samples for the lactose intolerance dataset and the hair color dataset), which prevents generating sufficient synthetic samples.

Given the limited sample size (at most 401 samples), our experiment may not fully capture the robustness of the scheme against machine learning-based MIAs in realistic scenarios, particularly for the lactose intolerance and hair color datasets. Nonetheless, the results provide valuable insights into privacy protection. The most realistic evaluation is conducted on the eye color dataset, which contains 401 samples, offering a scenario closer to real-world settings.

#### 7.2.4 Time Complexity Evaluation.

To evaluate the computational efficiency of each method, we measure the time required to generate privacy-preserving datasets across a range of SNP counts. This metric is used to compare our approach with Local Differential Privacy (LDP)[25], DPSyn[27], PrivBayes [54], and GAN [51], using the lactose intolerance dataset as a benchmark. For each configuration, we repeat the experiment 10 times and report the average runtime to reduce the effect of variability. By varying the number of SNPs, we assess how the time consumption of each method scales with data dimensionality, thereby revealing their practicality for large-scale genomic data sharing.

### 7.3 Experiment Setup

Local differential privacy (LDP) [25] and the proposed scheme cannot be compared directly since our scheme perturbs the data at the dataset level, while LDP perturbs each individual sample independently. To bridge this difference, we adopt the effective-ϵ formulation [22], which maps the dataset-level privacy budget in our scheme to an equivalent per-SNP value. This allows consistent and interpretable comparisons of privacy–utility trade-offs across all methods, including LDP [25], GAN-AG [51], DPSyn [27], and PrivBayes [54].

Unless otherwise specified, all experiments use $\epsilon_{e}\in \{1,2,3,4,5\}$. In GWAS studies, SNPs with p-values below α=0.05 are treated as significant, and the threshold is adjusted by a tolerance factor α/0.8=0.0625 following the relaxed verification criterion in Section 4.1. During validation, SNPs remaining significant under this relaxed threshold are considered retained. To evaluate performance against injected errors, we vary the error rate δ∈[0,1]. For robustness, each experiment is repeated ten times, and we report the mean performance with 95% confidence intervals shown as shaded regions in the plots. For neural networks used in membership inference attacks (MIAs), we adopt a feed-forward network with layer sizes 512, 128, 32, and 1, respectively, and LeakyReLU activations.

### 7.4 GWAS Outcome Validation

In this section, we evaluate how well our scheme supports reproducibility and error detection in genome-wide association studies (GWAS). The goal is to determine whether verifiers can reliably identify errors in reported outcomes using the shared privacy-preserving datasets. We compare our approach with both the local differential privacy (LDP) baseline [25] and several synthesis-based approaches, including GAN-AG [51], DPSyn [27], and PrivBayes [54]. A higher SNP retention-rate difference between the error-free and error-injected settings indicates better error detectability and, consequently, stronger validation performance.

#### 7.4.1 Comparison with LDP [25].

**Figure 2:**
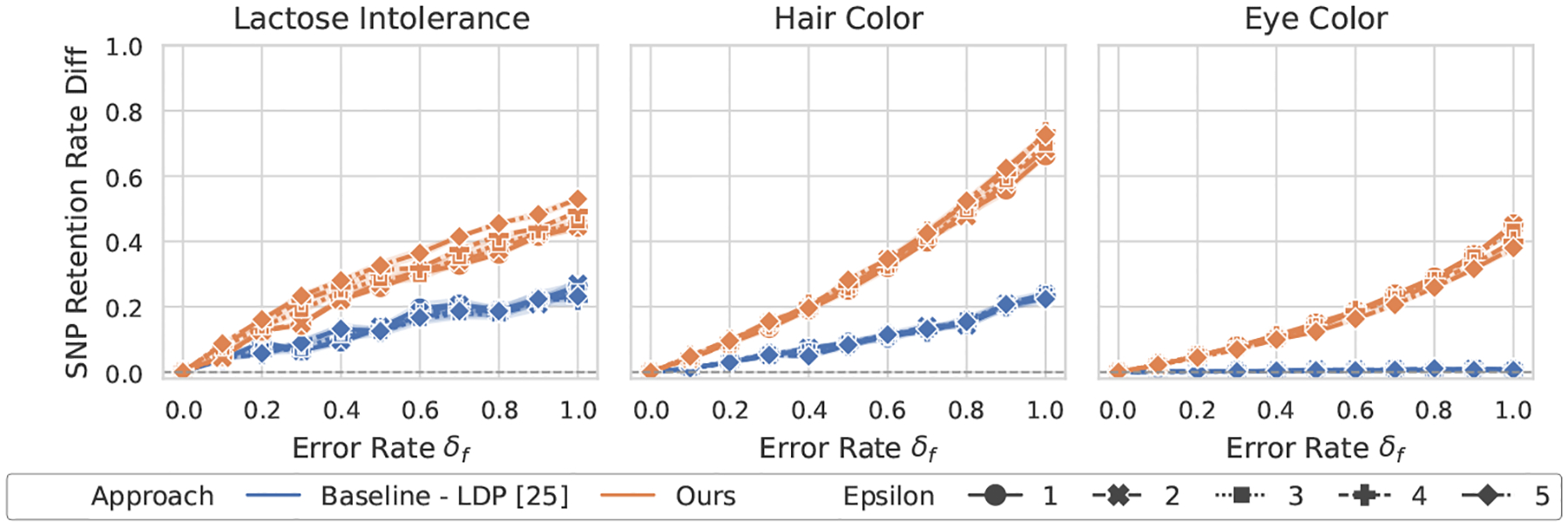
Performance of GWAS outcome validation for the $\chi^{2}$ test against flipping errors between ours and LDP [25].

**Figure 3:**
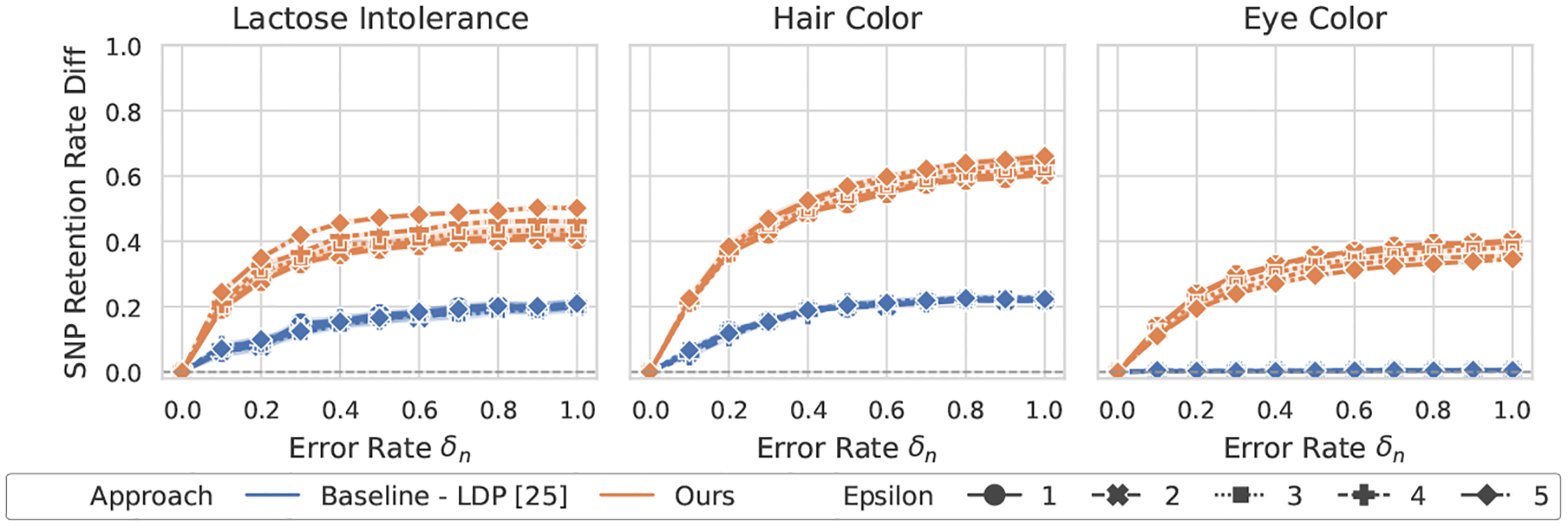
Performance of GWAS outcome validation for the $\chi^{2}$ test against noise errors between ours and LDP [25].

**Figure 4:**
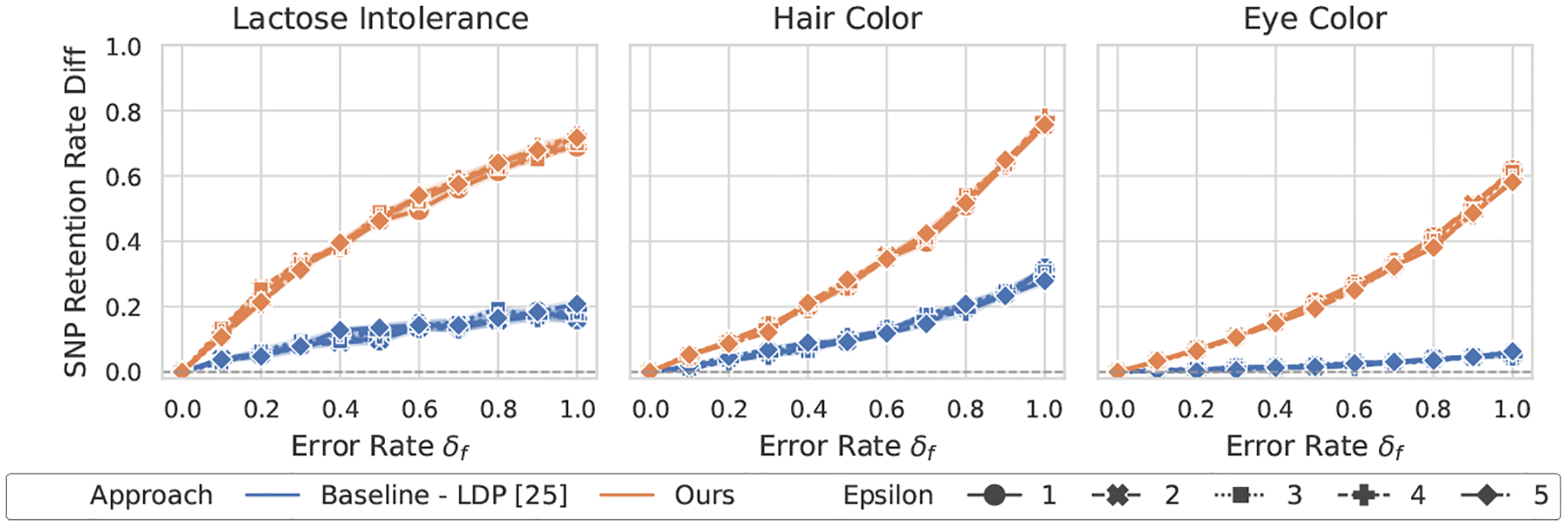
Performance of GWAS outcome validation for the odds ratio test against flipping errors between ours and LDP [25].

**Figure 5:**
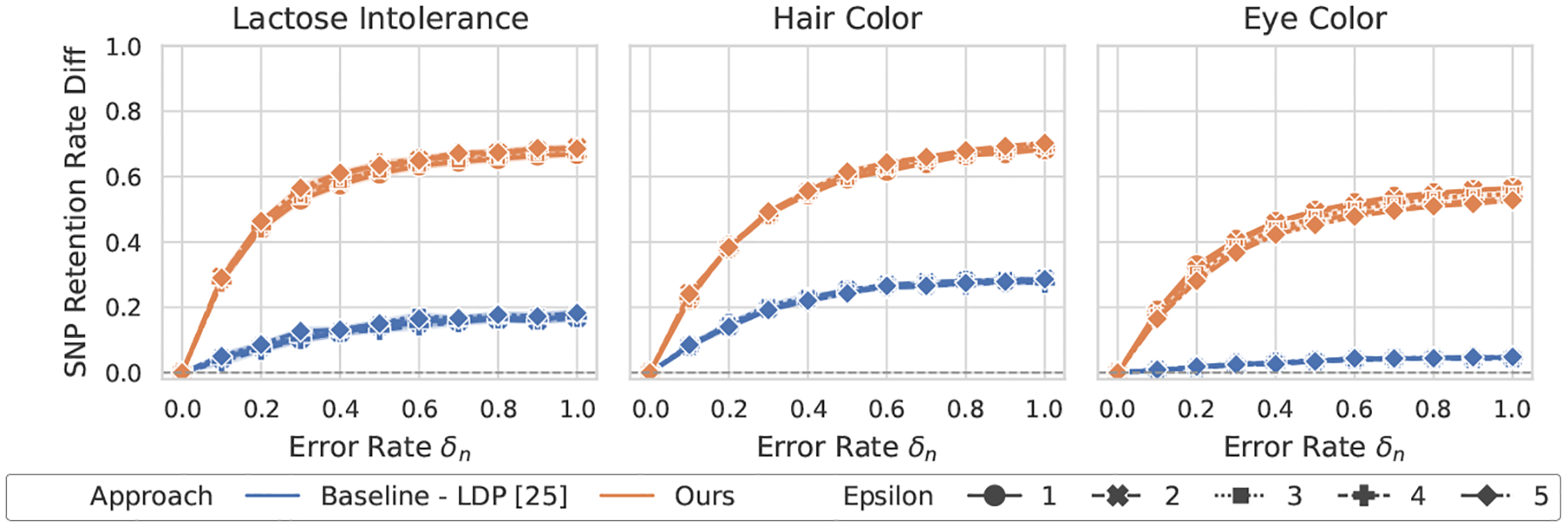
Performance of GWAS outcome validation for the odds ratio test against noise errors between ours and LDP [25].

Figures 2–5 show the overall performance of our method and LDP across different privacy levels. Our scheme consistently achieves higher SNP retention-rate differences than LDP [25]. This demonstrates stronger capability in detecting both flipping and noise errors. In the lactose intolerance and hair color datasets, our method shows clear differences when errors occur, with larger error rates leading to greater separation. The retention-rate difference ranges from about 0.4 (e.g., lactose intolerance under noise errors) to nearly 0.8 (e.g., hair color under flipping errors). These results confirm that our approach reliably detects small errors in both cases. In contrast, for LDP, the maximum difference at an error rate of 1.0 rarely exceeds 0.2.

In the larger eye color dataset, LDP shows almost no change in SNP retention rate across error levels, suggesting that it fails to identify erroneous results. Our method is slightly less sensitive in this dataset but still shows visible differences whenever errors occur. Overall, our scheme provides stronger and more interpretable GWAS validation than LDP under practical privacy budgets.

#### 7.4.2 Comparison with Synthesis-based Approaches.

We also implemented synthesis-based approaches, including the GAN approach [51], DPSyn [27], and PrivBayes [54], for comparison. However, we encountered significant limitations during their implementation.

DPSyn and PrivBayes depend on modeling internal correlations between features (SNPs in this context). Their computational cost increases non-linearly with dimensionality, which restricts their use to datasets containing at most 140 SNPs (see Section 7.7). This scale is far below the requirements of real GWAS datasets.

The GAN-AG model can complete dataset generation but suffers from severe utility loss (see Appendix G). In the lactose intolerance dataset, 51 of the 60 generated samples were identical, indicating that the model failed to capture the necessary variation. Across all datasets, the observed retention rate differences were close to zero, meaning that these synthetic datasets provide no meaningful signal for detecting GWAS errors.

Overall, synthesis-based approaches are impractical for GWAS outcome validation at realistic scales. Our method, in contrast, remains robust and reliable, consistently outperforming both LDP and synthesis-based methods in error detection while maintaining privacy and data utility.

#### 7.4.3 Evaluation under Non-Public (Noisy) MAF Release.

As discussed in Section 6.3, our default setting assumes that MAF statistics are publicly available under the NIH Genomic Data Sharing (GDS) policy. However, in scenarios where MAFs cannot be publicly released, e.g., due to access restrictions or additional privacy requirements, verifiers must rely on noisy, differentially private versions of these statistics. To simulate such cases and evaluate end-to-end privacy, we consider an alternative configuration in which both the shared dataset and the MAFs are protected under differential privacy.

In this configuration, the dataset is perturbed using our proposed dataset-sharing approach parameterized by $\epsilon_{e}$, while the MAFs are released using the Laplace mechanism parameterized by $\epsilon_{m}$. According to the composability property of differential privacy [17], if the dataset and the statistics are protected under privacy budgets $\epsilon_{e}$ and $\epsilon_{m}$, respectively, their joint release satisfies ($\epsilon_{e}+\epsilon_{m}$)-differential privacy. We implemented this configuration and performed GWAS outcome validation under these combined privacy budgets by fixing $\epsilon_{e}=1$ and varying $\epsilon_{m}\in 0.1,0.5,1.0$. As shown in Figure 7, PROVGEN maintains consistent validation accuracy across all datasets, with only a slight reduction compared to the public MAF case. These results demonstrate that PROVGEN continues to reliably detect GWAS errors even when both the dataset and the MAF statistics are protected by differential privacy.

### 7.5 Data Fidelity

Beyond evaluating GWAS reproducibility, we compared the performance of our scheme with LDP using the fidelity metrics introduced in Section 7.2.2. The results in Table 4 show that our scheme achieves consistently lower mean and variance errors than LDP across all datasets. These results indicate that the statistical characteristics of the original data are better preserved under our perturbation mechanism at the same privacy level as LDP.

For sample error and point error, LDP performs better in most cases. When combined with our advantage in mean and variance fidelity, this outcome suggests that our approach applies stronger perturbation at the individual-value level. The added noise introduces more variation in specific entries while maintaining global statistical consistency. In contrast, LDP perturbs each record independently with limited overall noise, resulting in smaller point-level deviations but weaker preservation of aggregate statistics.

We also compared our method against PrivBayes [54] and DPSyn [27] under a 100-SNP setting to assess performance on smaller datasets. As discussed in Section 7.3, these synthesis-based methods cannot scale to larger datasets. To ensure fairness, only 100 SNPs from each dataset are used for this comparison. Detailed results are provided in Appendix I.

### 7.6 Robustness Against Membership Inference Attacks

We evaluated the robustness of our scheme against membership inference attacks (MIAs) in comparison with LDP across three datasets. Table 5 summarizes the maximum attack power observed in each setting, where a higher value indicates greater privacy risk. Note that additional results covering a wider range of $\epsilon_{e}$ values are provided in Appendix H due to space constraints.

Our scheme achieves lower maximum attack power than LDP in the eye color and lactose intolerance datasets, showing stronger resistance to MIAs across all $\epsilon_{e}$ values. In contrast, LDP remains consistently more vulnerable. For the hair color dataset, both methods exhibit similar performance when $\epsilon_{e}\geq 3$, with our approach recording 75% attack power under SVM and 83.3% under the Hamming distance test. This higher vulnerability is primarily attributed to the small sample size and high feature dimensionality of this dataset, which favor dimension-aware classifiers such as SVM.

**Figure 6:**
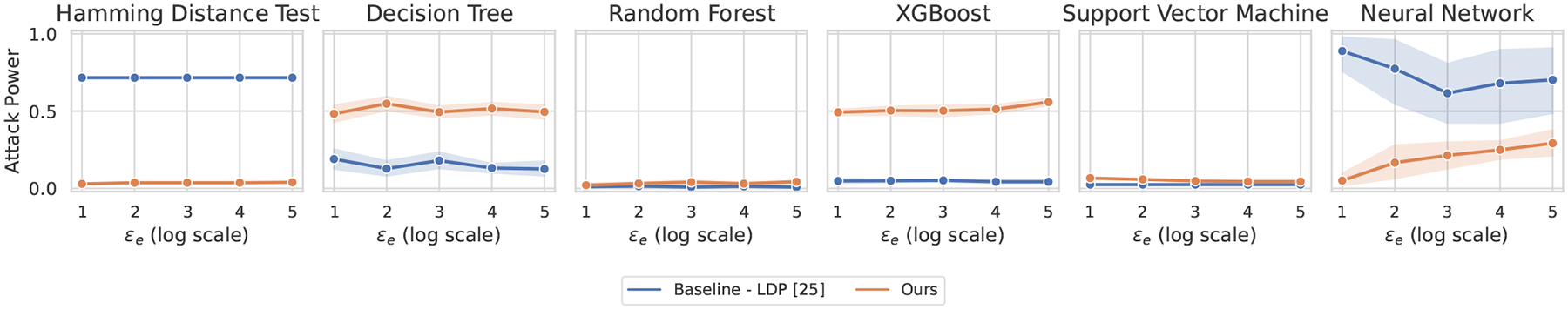
Comparison of our approach and local differential privacy (LDP) [25] against MIAs on the eye color dataset. Our scheme maintains low attack power for $\epsilon_{e}<5$, while LDP remains vulnerable to Hamming distance and neural network attacks.

**Figure 7:**
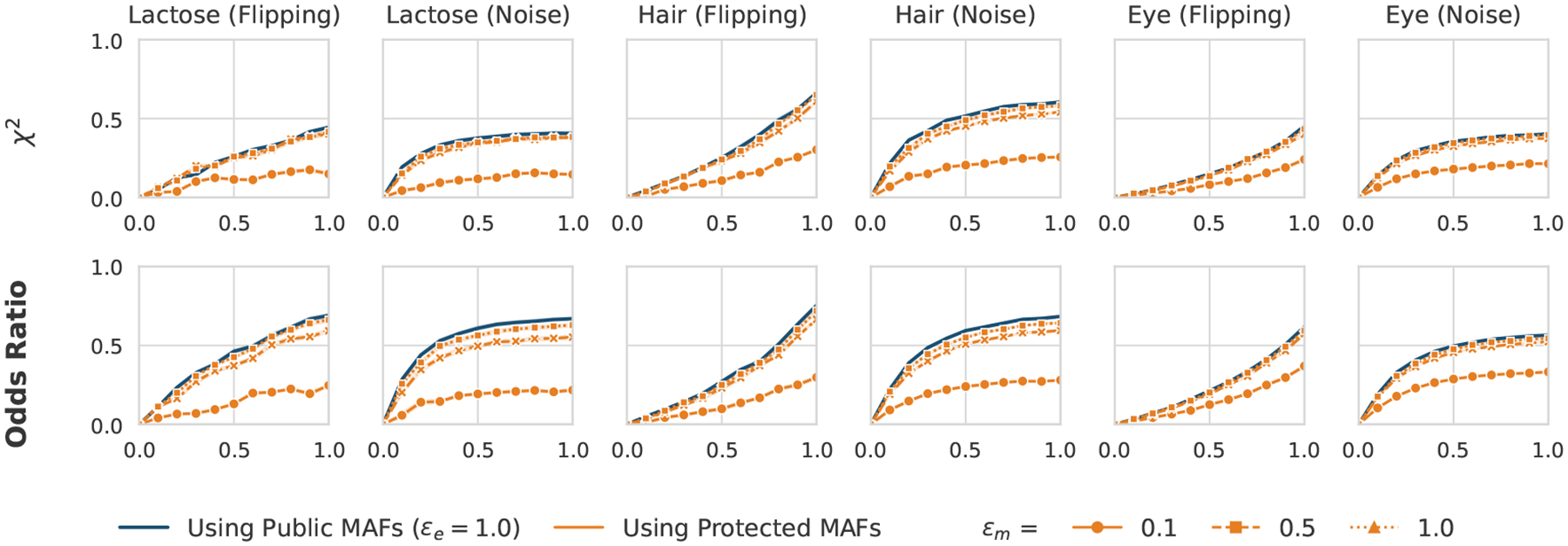
GWAS outcome validation under combined privacy budgets ($\epsilon_{e}=1,\epsilon_{m}\in \{0.1,0.5,1.0\}$) for the $\chi^{2}$ and odds ratio tests.

Detailed per-dataset analysis further supports this observation. In the eye color dataset (Figure 6), LDP exhibits high attack power under both the Hamming distance test (82.7% across all $\epsilon_{e}$ values) and neural networks (up to 92.7% with large variance), whereas our method remains resistant, with attack power around 50% for decision tree and XGBoost attacks. For the lactose intolerance and hair color datasets (Appendix H), LDP achieves higher attack power in most cases; however, the instances where our scheme performs worse are generally close to 50%, comparable to random guessing. The only exception is the SVM attack on the hair color dataset, where our scheme reaches 75% attack power. Nevertheless, this value remains lower than the most effective attack type Hamming distance test (83.3%) indicating that our approach still provides robust protection.

Overall, our method demonstrates stronger resilience to membership inference attacks under realistic privacy budgets while maintaining high data utility.

### 7.7 Time Complexity

We evaluated the time efficiency of our scheme against vanilla XOR [24], LDP [25], DPSyn [27], and PrivBayes [54]. All experiments were conducted on an Intel(R) Xeon(R) Silver 4416+ 20-core HPC server. The number of samples was fixed at 100, while the number of SNPs was varied to simulate realistic genomic data generation. The original XOR mechanism can only generate up to 10 SNPs within a one-hour time limit, and synthetic data generators such as DPSyn and PrivBayes complete generation for about 100 SNPs. In contrast, our proposed method scales efficiently to the full 28,000-SNP eye color dataset. Although LDP achieves the fastest runtime, it is impractical for GWAS validation due to weak error detection performance (Section 7.4.1) and high vulnerability to membership inference attacks (Section 7.6). Since the number of SNPs involved in typical GWAS validation rarely exceeds this scale, our method can effectively support realistic genomic studies. Overall, PROVGEN achieves the best balance between computational efficiency, validation accuracy, and privacy protection.

## 8 Limitations and Future Work

In this paper, we propose a novel scheme for sharing genomic datasets in a privacy-preserving manner, specifically for GWAS outcome validation. We efficiently adapted the XOR mechanism to generate binary datasets while preserving correlations with the help of published Minor Allele Frequencies (MAFs). Our approach demonstrates superiority in detecting GWAS outcome errors, maintaining data fidelity, and providing robustness against membership inference attacks.

While our approach is specifically optimized for enhancing GWAS reproducibility, it has certain limitations. It may not generalize well to other genomic studies, such as transcriptome-wide association studies, genetic epidemiology, or gene-environment interaction analyses. Additionally, our method does not explicitly address scenarios where malicious researchers fabricate datasets to report false results. However, the likelihood of such misconduct is low, as ethical risks and potential career repercussions serve as strong deterrents.

Despite these limitations, our method remains highly effective within its intended scope, significantly improving the reproducibility and utility of GWAS outcomes. Moving forward, we will explore strategies to further optimize privacy and usability in practical genomic research settings. Future work will also focus on integrating dataset fingerprinting techniques to enhance accountability and strengthen privacy assurances in genomic data sharing.

## Figures and Tables

**Figure 8:**
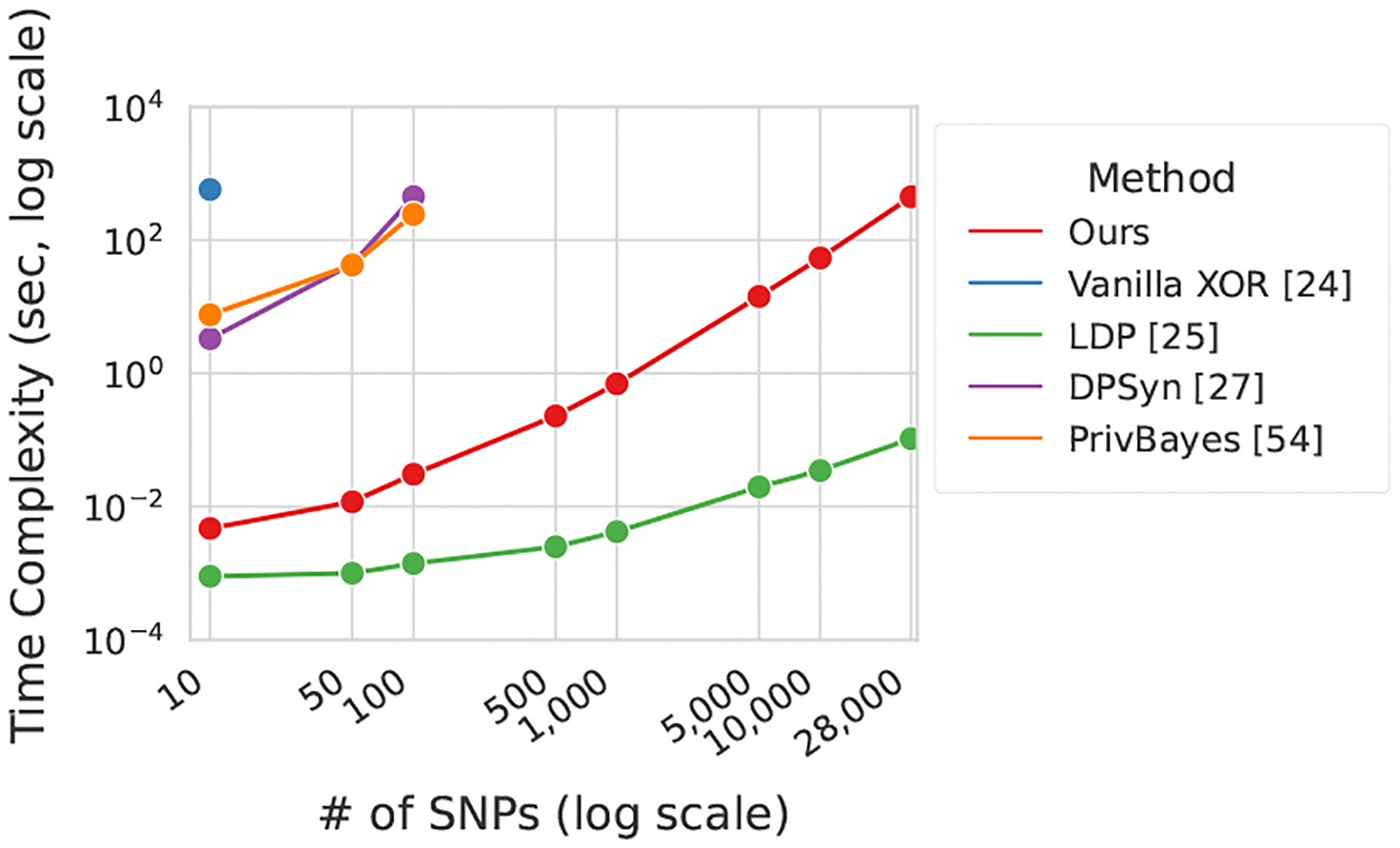
Time complexity.

**Table 1:** A contingency table.

	Genotype			
	0	1	2	Total
Case	$S_{0}$	$S_{1}$	$S_{2}$	S
Control	$R_{0}$	$R_{1}$	$R_{2}$	R
Total	$N_{0}$	$N_{1}$	$N_{2}$	N

**Table 2:** Conversion between SNP values and binary format.

Genomic Value	Binary Format
0	00
1	01
2	11

**Table 3:** Comparison of noise generation methods.

	considered PDF	sampling approach	privacy guarantee
[24]	$B\sim f_{ℬ}(B)$ in (1), matrix format	Hamiltonian Monte Carlo	Thm. 3.3
Ours	$B_{u}\sim \text{Pr}\left[B_{u}=1\right]$ in (8), scalar format	independent Bernoulli draws	Thm. 6.2, a suffcicent condition for Thm. 3.3

**Table 4:** Data fidelity comparisons across datasets for our approach versus LDP [25]. Confidence intervals for all results are very small and thus omitted. Outcomes with superior (lower) results are highlighted in bold.

Dataset	$\epsilon_{e}$	Sample Error		Point Error		Mean Error		Variance Error	
		LDP [25]	Ours	LDP [25]	Ours	LDP [25]	Ours	LDP [25]	Ours
Eye Color	1	**0.3110**	0.3829	**0.2222**	0.3405	0.2324	**0.0006**	0.2549	**0.0325**
	2	**0.3111**	0.3681	**0.2223**	0.3309	0.2324	**0.0006**	0.2549	**0.0333**
	3	**0.3110**	0.3509	**0.2222**	0.3191	0.2324	**0.0006**	0.2548	**0.0346**
	4	**0.3111**	0.3321	**0.2222**	0.3054	0.2325	**0.0007**	0.2549	**0.0360**
	5	**0.3112**	0.3126	**0.2223**	0.2905	0.2325	**0.0007**	0.2550	**0.0372**
Hair Color	1	**0.3068**	0.3832	**0.2221**	0.3455	0.2204	**0.0041**	0.2416	**0.0396**
	2	**0.3071**	0.3443	**0.2223**	0.3178	0.2201	**0.0043**	0.2414	**0.0386**
	3	0.3066	**0.3033**	**0.2220**	0.2855	0.2199	**0.0050**	0.2413	**0.0375**
	4	0.3071	**0.2663**	**0.2222**	0.2543	0.2203	**0.0058**	0.2418	**0.0359**
	5	0.3069	**0.2355**	**0.2223**	0.2269	0.2199	**0.0063**	0.2412	**0.0338**
Lactose Intolerance	1	**0.3086**	0.4352	**0.2221**	0.3900	0.2200	**0.0042**	0.2149	**0.0609**
	2	**0.3087**	0.3901	**0.2221**	0.3576	0.2199	**0.0038**	0.2152	**0.0606**
	3	**0.3092**	0.3411	**0.2224**	0.3191	0.2204	**0.0047**	0.2156	**0.0587**
	4	0.3089	**0.2945**	**0.2223**	0.2797	0.2201	**0.0060**	0.2151	**0.0549**
	5	0.3089	**0.2536**	**0.2223**	0.2433	0.2203	**0.0073**	0.2155	**0.0496**

**Table 5:** Comparison of maximum attack power against MIAs. Better results are marked in bold. When both methods achieve the same value, both are bolded.

Dataset	Approach	$\epsilon_{e}$				
		1	2	3	4	5
Eye Color	Baseline - LDP [25]	0.927	0.907	0.827	0.878	0.870
	Ours	**0.525**	**0.564**	**0.521**	**0.541**	**0.564**
Hair Color	Baseline - LDP [25]	0.833	0.833	**0.833**	**0.833**	**0.833**
	Ours	**0.750**	**0.750**	**0.833**	**0.833**	**0.833**
Lactose Intolerance	Baseline - LDP [25]	0.850	0.867	0.867	0.883	0.867
	Ours	**0.508**	**0.550**	**0.558**	**0.642**	**0.767**

**Figure 9:**
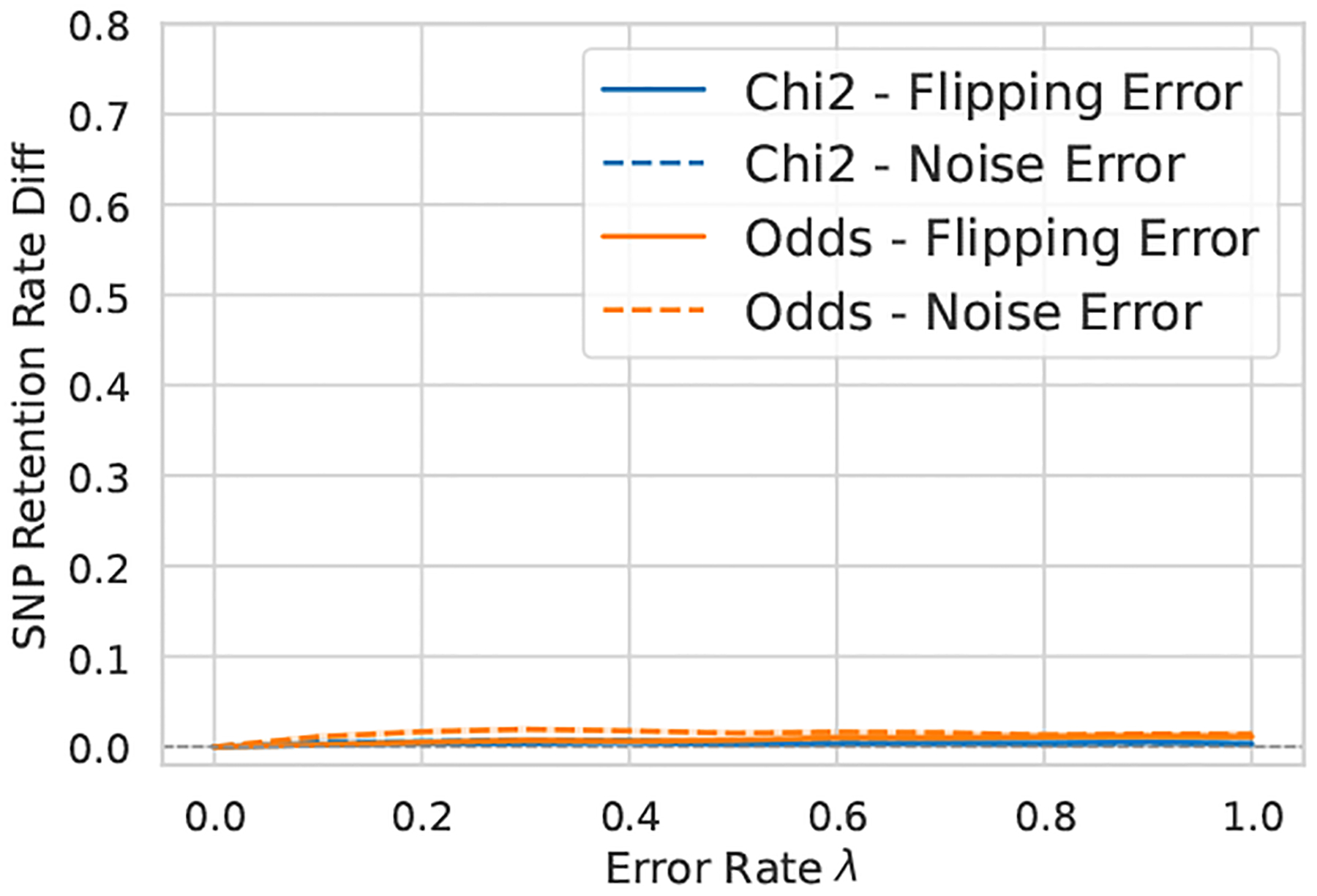
Performance of the GAN approach [51] for GWAS outcome validation.

**Figure 10:**
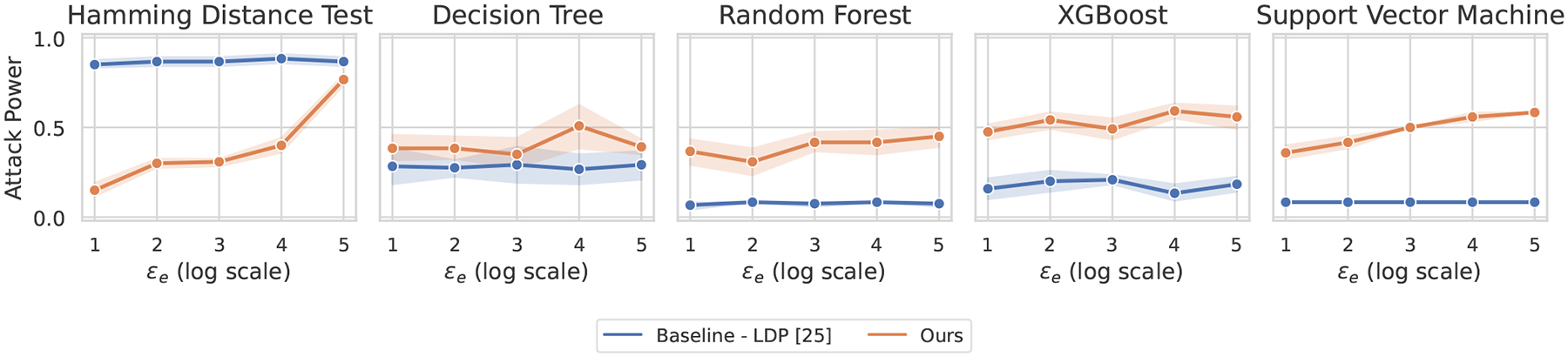
Comparison of robustness of our approach and local differential privacy (LDP) [25] against different membership inference attacks on the lactose intolerance dataset.

**Figure 11:**
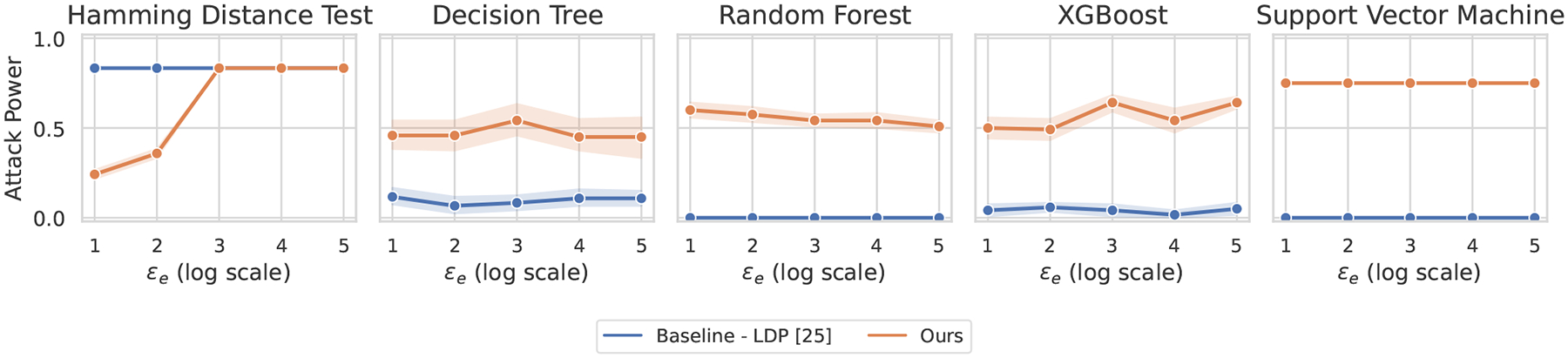
Comparison of robustness of our approach and local differential privacy (LDP) [25] against different membership inference attacks on the hair color dataset.

**Figure 12:**
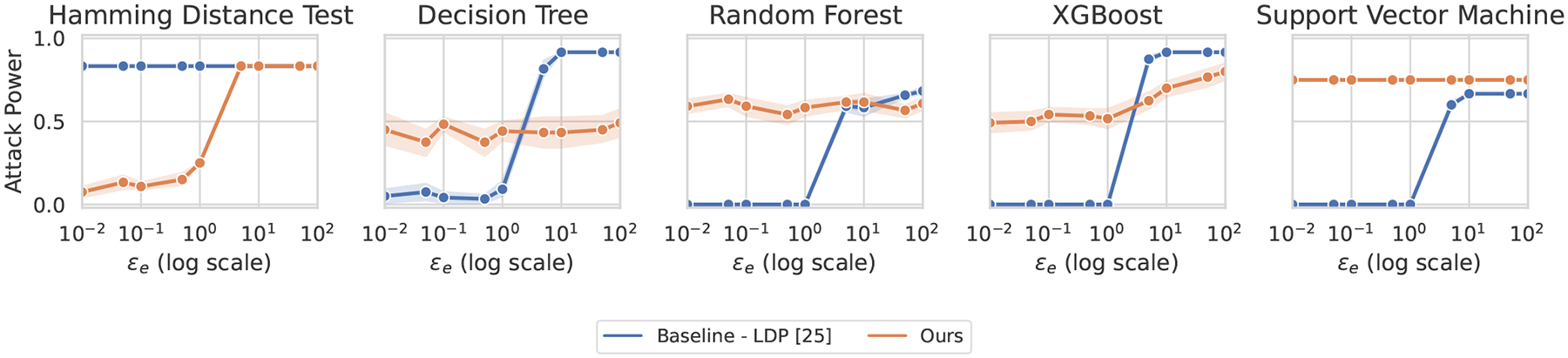
Comparison of the robustness of our approach and local differential privacy (LDP) [25] against multiple membership inference attacks on the hair color dataset, evaluated over a wide privacy range $\epsilon_{e}\in \left[10^{-2},10^{2}\right]$.

**Figure 13:**
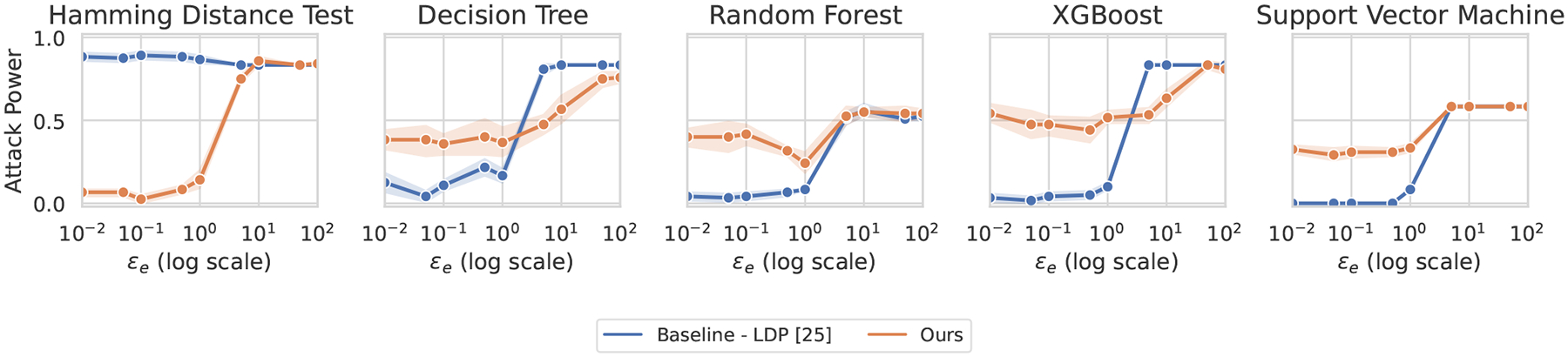
Comparison of robustness of our approach and local differential privacy (LDP) [25] against different membership inference attacks on the lactose intolerance dataset, evaluated over a wide privacy range $\epsilon_{e}\in \left[10^{-2},10^{2}\right]$.

**Figure 14:**
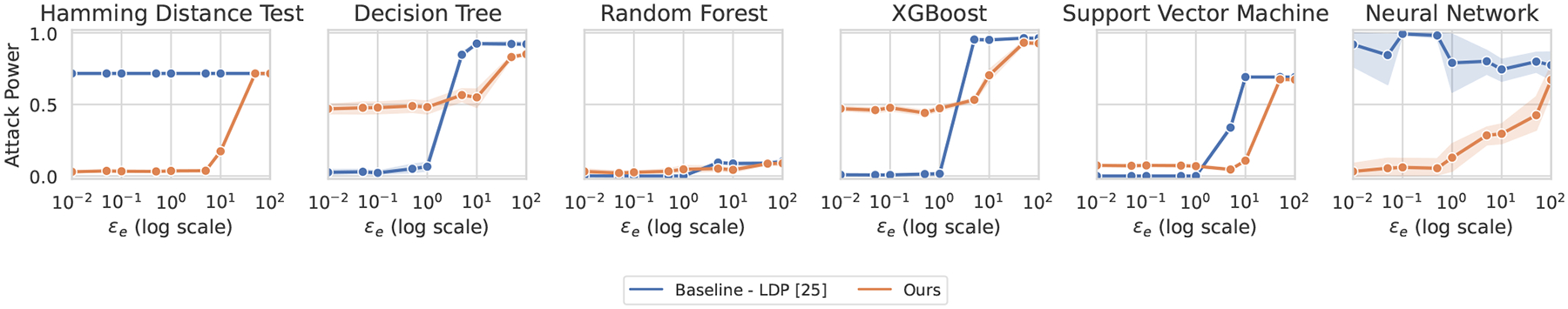
Comparison of robustness of our approach and local differential privacy (LDP) [25] against different membership inference attacks on the eye color dataset, evaluated over a wide privacy range $\epsilon_{e}\in \left[10^{-2},10^{2}\right]$.

## A Appendix

### B Symbols and Notations

We show frequent symbols and notations used in the paper in Table 6.

**Table 6:** Symbols and notations.

Notations	Descriptions
n	The number of individuals in the dataset D
m	The number of SNPs in the dataset D
$D^{b}$	The binarized version of D
${\tilde{D}}^{b}$	The perturbed (binarized) dataset from Stage 1
${\overset{ˆ}{D}}^{b}$	The utility-restored (binarized) dataset from Stage 2
$D^{\prime }$	The output dataset
$\epsilon_{e}$	Effective privacy budget for each SNP

### C Statistical Tests for GWAS Evaluation

#### $\chi^{2}$ Test.

The $\chi^{2}$ test assesses whether the observed frequencies of SNP values in case and control groups differ significantly from expected values under the null hypothesis of no association. A larger $\chi^{2}$ value indicates a greater deviation from expectation, implying a stronger association with the phenotype or disease.

#### Odds Ratio Test.

The odds ratio (OR) test evaluates the strength of association between an SNP and a trait. The OR is calculated as:

(12)
$$OR=\frac{\left(S_{1}+S_{2}\right)/\left(R_{1}+R_{2}\right)}{S_{0}/R_{0}}=\frac{R_{0}\left(S_{1}+S_{2}\right)}{S_{0}\left(R_{1}+R_{2}\right)}.$$

To assess significance, we compute the 95% confidence interval as exp(ln(OR)±1.96×SE(ln(OR))), where:

$$SE(\text{ln}\;(OR))=\sqrt{\frac{1}{S_{1}+S_{2}}+\frac{1}{S_{0}}+\frac{1}{R_{1}+R_{2}}+\frac{1}{R_{0}}}.$$

We then compute the z-value as $\frac{\text{ln}\;(OR)}{SE(\text{ln}\;(OR))}$ and derive the p-value from the standard normal distribution.

### D Proof of Theorem 6.2

Proof. In the first step, we denote

$${𝒮}_{u}=\left\{b|b_{u}=1,b_{v}\in \{0,1\},\forall v\in \{1,2,\cdots ,NP\},v\neq u\right\}.$$

Then, we bound the marginal probability of each noise bit taking value 1 as follows

$$\text{Pr}\left[b_{u}=1\right]=\sum_{b\in {𝒮}_{u}}C(𝚷)\text{exp}\left\{b^{T}𝚷b\right\}$$

$$=\frac{\sum_{b\in {𝒮}_{u}}\text{exp}\left\{b^{T}𝚷b\right\}}{\sum_{b\in 𝒮}\text{exp}\left\{b^{T}𝚷b\right\}}$$

$$\overset{\left(a\right)}{=}\frac{\sum_{b\in {𝒮}_{u}}\text{exp}\left\{b^{T}𝚷b\right\}}{\sum_{b\in {𝒮}_{u}}\text{exp}\left\{b^{T}𝚷b\right\}+\sum_{b\in \bar{{𝒮}_{u}}}\text{exp}\left\{b^{T}𝚷b\right\}}$$

$$=\frac{1}{1+\frac{\sum_{b\in \bar{{𝒮}_{u}}}\text{exp}\left\{b^{T}𝚷b\right\}}{\sum_{b\in {𝒮}_{u}}\text{exp}\left\{b^{T}𝚷\right\}}}$$

$$\overset{\left(b\right)}{=}\frac{1}{1+\frac{\sum_{b\in \bar{{𝒮}_{u}}}\text{exp}\left\{b^{T}𝚷b\right\}}{\sum_{b\in \bar{{𝒮}_{u}}}\text{exp}\left\{{\left(b+j_{u}\right)}^{T}𝚷\left(b+j_{u}\right)\right\}}}$$

$$\overset{\left(c\right)}{\leq }\frac{1}{1+{\text{min}}_{b\in \bar{{𝒮}_{u}}}\frac{\text{exp}\left\{b^{T}𝚷b\right\}}{\text{exp}\left\{{\left(b+j_{u}\right)}^{T}𝚷\left(b+j_{u}\right)\right\}}}$$

$$\leq \frac{1}{1+{\text{max}}_{b\in \bar{{𝒮}_{u}}}\text{exp}\left\{2j_{u}^{T}𝚷b+{𝚷}_{u,u}\right\}}$$

where in (a), $\bar{{𝒮}_{u}}$ is the complementary set of ${𝒮}_{u}$, i.e.,

$$\bar{{𝒮}_{u}}=\left\{b|b_{u}=0,b_{v}\in \{0,1\},\forall v\in \{1,2,\cdots ,NP\},v\neq u\right\}.$$

(b) is because by defining the one-hot vector

$$j_{u}\in \{0,1{\}}^{nP\times 1}$$

that only has 1 at the uth position, and 0 at all the other positions. Then, $\forall b\in \bar{{𝒮}_{u}}$, we have $b+j_{u}\in {𝒮}_{u}$. (*c*) is because $\frac{\sum x_{i}}{\sum y_{i}}\geq {\text{min}}_{i}\frac{x_{i}}{y_{i}}$ for positive sequences $x_{i}$ and $y_{i}$, and in $(c)\;{𝚷}_{u,u}$ represents the entry of 𝚷 in the uth row and uth column.

In step 2, we proceed to calculate the maximum value, i.e.,

$${\text{max}}_{b\in \bar{{𝒮}_{u}}}\text{exp}\left\{2j_{u}^{T}𝚷b+{𝚷}_{u,u}\right\}=\text{exp}\left\{{𝚷}_{u,u}+{\text{max}}_{b\in \bar{{𝒮}_{u}}}2j_{u}^{T}𝚷b\right\}.$$

In particular, we observe that $j_{u}^{T}𝚷$ represents the uth row of 𝚷, thus ${\text{max}}_{b\in \bar{{𝒮}_{u}}}j_{u}^{T}𝚷b$ corresponds to the summation of all positive values in the uth row of 𝚷 except for ${𝚷}_{u,u}$ (since $b_{u}=0$). We denote $\kappa_{u}=2\times Sum\left({𝚷}_{u}\right)-{𝚷}_{u,u}$.

In step 3, we prove that the probability ratio of the outputs of the efficient genomic dataset perturbation is bounded by exp(ϵ). W.l.o.g., suppose D and $D^{\prime }$ only differ by the SNP sequence of the first individual, and let d and $d^{\prime }$ be the encoded SNP sequences of the first individual in D and $D^{\prime }$, respectively.

$$\text{ln}\left(\frac{\prod_{u}\text{Pr}\left(d_{u}⊕B_{u}=O_{u}\right)}{\prod_{u}\text{Pr}\left(d_{u}^{\prime }⊕B_{u}^{\prime }=O_{u}\right)}\right)$$

$$=\sum_{u}\text{ln}\frac{\text{Pr}\left(B_{u}=O_{u}⊕d_{u}\right)}{\text{Pr}\left(B_{u}^{\prime }=O_{u}⊕d_{u}^{\prime }\right)}$$

$$=\sum_{u}\text{ln}\;\frac{\text{Pr}{\left(B_{u}=1\right)}^{O_{u}⊕d_{u}}{\left(1-\text{Pr}\left(B_{u}=1\right)\right)}^{1-\left(O_{u}⊕d_{u}\right)}}{\text{Pr}{\left(B_{u}^{\prime }=1\right)}^{O_{u}⊕d_{u}^{\prime }}{\left(1-\text{Pr}\left(B_{u}^{\prime }=1\right)\right)}^{1-\left(O_{u}⊕d_{u}^{\prime }\right)}}$$

$$=\sum_{u}\left[\left(O_{u}⊕d_{u}\right)-\left(O_{u}⊕d_{u}^{\prime }\right)\right]\;\text{ln}\;\text{Pr}\left(B_{u}=1\right)+\left[\left(O_{u}⊕d_{u}^{\prime }\right)-\left(O_{u}⊕d_{u}\right)\right]\;\text{ln}\left(1-\text{Pr}\left(B_{u}=1\right)\right)$$

$$=\left(\sum_{\left\{u:K_{u}>||\lambda (𝚯)||\right\}}\left[\left(O_{u}⊕d_{u}\right)-\left(O_{u}⊕d_{u}^{\prime }\right)\right]\;\text{ln}\;\text{Pr}\left(B_{u}=1\right)+\left[\left(O_{u}⊕d_{u}^{\prime }\right)-\left(O_{u}⊕d_{u}\right)\right]\;\text{ln}\left(1-\text{Pr}\left(B_{u}=1\right)\right)\right)$$

$$+\left(\sum_{\left\{u:\kappa_{u}\leq \|\lambda (𝚯)\|\right\}}\left[\left(O_{u}⊕d_{u}\right)-\left(O_{u}⊕d_{u}^{\prime }\right)\right]\;\text{ln}\;\text{Pr}\left(B_{u}=1\right)+\left[\left(O_{u}⊕d_{u}^{\prime }\right)-\left(O_{u}⊕d_{u}\right)\right]\;\text{ln}\left(1-\text{Pr}\left(B_{u}=1\right)\right)\right)$$

$$\overset{\left(a\right)}{=}\sum_{\left\{u:\kappa_{u}\leq \|\lambda (\Theta )\|\right\}}\left[\left(O_{u}⊕d_{u}\right)-\left(O_{u}⊕d_{u}^{\prime }\right)\right]\;\text{ln}\frac{1}{1+\text{exp}\left(\kappa_{u}\right)}+\left[\left(O_{u}⊕d_{u}^{\prime }\right)-\left(O_{u}⊕d_{u}\right)\right]\;\text{ln}\frac{\text{exp}\left(\kappa_{u}\right)}{1+\text{exp}\left(\kappa_{u}\right)}$$

$$=\sum_{\left\{u:\kappa_{u}\leq \|\lambda (\Theta )\|\right\}}\left[\left(O_{u}⊕d_{u}\right)-\left(O_{u}⊕d_{u}^{\prime }\right)\right]\times \left(\text{ln}\frac{1}{1+\text{exp}\left(\kappa_{u}\right)}-\text{ln}\frac{\text{exp}\left(\kappa_{u}\right)}{1+\text{exp}\left(\kappa_{u}\right)}\right)$$

$$=\sum_{\left\{u:\kappa_{u}\leq \|\lambda (\Theta )\|\right\}}\left[\left(O_{u}⊕d_{u}\right)-\left(O_{u}⊕d_{u}^{\prime }\right)\right]\;\text{ln}\frac{1}{\text{exp}\left(\kappa_{u}\right)}$$

$$\overset{\left(b\right)}{=}\sum_{\left\{u:\kappa_{u}\leq \|\lambda (\Theta )\|\right\}}\left|2O_{u}-1\left\|d_{u}^{\prime }-d_{u}\right\|\kappa_{u}\right|$$

$$\overset{\left(c\right)}{<}s_{f}\|\lambda (\Pi ){\|}_{2}\leq s_{f}(\|\lambda (\Theta ){\|}_{2}+\sum_{i=1}^{n-1}\sum_{j=i+1}^{n}{\left\|\lambda (\Lambda_{i,j})\right\|}_{2}),$$

where (a) is because the summation is 0 for $\left\{u:\kappa_{u}>\|\lambda (\Theta )\|\right\}$, (b) is because u⊕v=(1-u)v+u(1-v) for binary u and v, and (c) is because the cardinity of set $\left\{u:\kappa_{u}\leq \|\lambda (𝚯)\|\right\}$ is at most $s_{f}$. According to (5), we can complete the proof. □

### E Further Details About the Post-Processing

We use the Minor Allele Frequencies (MAFs) published in the research findings, denoted as ${ℳ}^{r}$, as a reference and calculate the MAFs in the noisy dataset ${\tilde{D}}^{b}$ as $\tilde{ℳ}$. We first convert these MAFs into binary distributions with percentages of 0’s and 1’s, denoted as $C_{j}$ for ${ℳ}_{j}^{r}$ and ${\tilde{𝒞}}_{j}$ for ${\tilde{ℳ}}_{j}$. Our goal is to adjust the distribution of 0’s and 1’s in ${\tilde{D}}^{b}$ so that the final dataset, denoted as ${\overset{ˆ}{D}}^{b}$, aligns its MAFs closely with the public MAFs.

First, we define the cost as $C_{pq}=|p-q|$ for the change from p to q, where p,q∈{0,1}, aiming to modify the dataset with the minimum total cost. We then construct an optimization problem at each position j in the encoded genomic dataset ${\tilde{D}}^{b}$. We consider $C_{j}^{\prime }$ and ${\tilde{C}}_{j}$ as two mass distributions at position j, aiming to find a transport plan $T^{𝒦\times 𝒦}$ that modifies the mass of $C_{j}^{\prime }$ to make it resemble ${\tilde{C}}_{j}$. The total cost is defined as:

(13)
$$\left\langle T,C\right\rangle =\sum_{p=0}^{1}\sum_{q=0}^{1}T_{pq}C_{pq},$$

where T is the transport plan and C is the matrix of costs.

The optimal transport is formulated as follows:

$$\underset{T}{\text{min}}\langle T,C\rangle$$

$$\text{s}.\text{t}.\;\sum_{q=0}^{1}T_{pq}=c_{pj}^{\prime \text{norm}}\forall p\in \left\{0,1\right\}$$

$$\sum_{p=0}^{1}T_{pq}={\tilde{c}}_{qj}^{\text{norm}}\forall q\in \left\{0,1\right\}$$

$$T_{pq}\geq 0\;\forall (p,q)\in \{0,1\}\times \{0,1\},$$

where ${\tilde{C}}_{kj}^{\text{norm}}$ and $C_{kj}^{\prime \text{norm}}$ are normalized as:

(14)
$${\tilde{C}}_{kj}^{\text{norm}}=\frac{{\tilde{c}}_{kj}}{\left|{\tilde{C}}_{j}\right|},C_{kj}^{\prime \text{norm}}=\frac{c_{kj}^{\prime }}{\left|C_{j}^{\prime }\right|},k\in \left\{0,1\right\}$$

Here, ${\tilde{c}}_{kj}$ and $c_{kj}^{\prime }$ are the counts of SNP value k at position j in $\tilde{C}$ and $C^{\prime }$, respectively. T essentially represents a joint mass distribution at each position $j\left(\sum_{p}\sum_{q}T_{pq}=1,\forall j\right)$ whose row- or column-wise marginalization is the marginal distribution of SNP taking value p or q at position j.

This one-dimensional optimization problem is solved using optimal transport (OT), a method in transportation theory aimed at minimizing the cost while transferring the distribution from one state to another. We use the existing Python package [20] to calculate this one-dimensional optimal transport, applying the formulated strategy to adjust SNP values based on $T_{pq}$, effectively transferring $\left\lfloor T_{pq}\times n\right\rfloor$ alleles from one category to another.

### F Ethical Considerations

In our study, we utilized existing public genomic datasets to extract inherent correlations between SNPs in the noise generation step (Section 6.2) and leveraged the published minor allele frequencies (MAFs) to perform utility restoration (Section 6.3). While these datasets are publicly accessible and have been previously cleared for use in research, we recognize the importance of addressing ethical considerations in our work.

In particular, we ensure that the use of these datasets aligns with their intended purposes as defined by the original data providers. Our methodologies and objectives are consistent with the terms under which these datasets were made public. Our approach, focuses on maintaining the anonymity integral to these datasets, avoids any attempts at re-identification and strictly follows protocols to prevent de-anonymization. Meanwhile, our work does not involve direct interaction with human participants, thus significantly reducing ethical risks commonly associated with primary data collection. We adhere to recognized standards for secondary data usage, and continually stay informed about ethical guidelines and best practices in genomic research to ensure ongoing compliance.

### G GWAS Outcome Validation Using GAN-AG

Figure 9 reports the GWAS outcome validation performance of the GAN-AG approach [51].

### H Details of the Experiment Results Against MIAs

We present detailed experimental results for the lactose intolerance and hair color datasets against multiple membership inference attacks in Figures 10 and 11. For lactose intolerance, both our scheme and LDP exhibit attack accuracies close to 50% across all classifiers, except under the Hamming distance test where LDP performs slightly worse at higher $\epsilon_{e}$ values. For hair color, our scheme shows stronger resistance under the Hamming distance test, while LDP performs slightly better under most machine learning attacks such as decision tree, random forest, and XGBoost. However, due to the dataset’s small sample size, these results are less representative of large-scale GWAS settings. We additionally evaluate a wider privacy range, $\epsilon_{e}\in \left[10^{-2},10^{2}\right]$, in Figures 12–14.

### I Additional Utility Comparison

In our analysis, we conduct a comprehensive utility comparison of our method against DPSyn [27] and PrivBayes [54] across three toy datasets, each comprising 100 SNPs, as shown in Figure 7.

**Table 7:** Comprehensive comparison in data fidelity across three 100-SNP toy datasets for our approach versus DPSyn [27] and PrivBayes [54]. Outcomes with superior results are highlighted in bold.

	Utility Metric	Sample Error			Mean Error			Point Error			Variance Error		
	Approach	DPSyn [27]	PrivBayes [54]	Ours	DPSyn [27]	PrivBayes [54]	Ours	DPSyn [27]	PrivBayes [54]	Ours	DPSyn [27]	PrivBayes [54]	Ours
Dataset	$\epsilon_{e}$												
Lactose Intolerance	1	0.8818	0.6052	**0.4866**	0.6107	0.2039	**0.1764**	0.6476	0.4775	**0.4233**	0.2973	0.1750	**0.1395**
	2	0.8818	0.5441	**0.4794**	0.6107	**0.1251**	0.1765	0.6476	0.4400	**0.4172**	0.2973	**0.1189**	0.1387
	3	0.8818	0.5256	**0.4705**	0.6107	**0.0972**	0.1764	0.6476	0.4276	**0.4097**	0.2973	**0.0963**	0.1395
	4	0.8818	0.5136	**0.4657**	0.6107	**0.0815**	0.1758	0.6476	0.4200	**0.4049**	0.2973	**0.0828**	0.1374
	5	0.8818	0.5085	**0.4590**	0.6107	**0.0789**	0.1756	0.6476	0.4184	**0.3974**	0.2973	**0.0807**	0.1362
Hair Color	1	0.6986	**0.4842**	0.5088	0.4193	**0.1292**	0.2646	0.5517	**0.4075**	0.4414	0.2233	**0.1053**	0.1736
	2	0.6986	**0.4572**	0.5014	0.4193	**0.0854**	0.2643	0.5517	**0.3917**	0.4358	0.2233	**0.0728**	0.1736
	3	0.6986	**0.4521**	0.4979	0.4193	**0.0698**	0.2646	0.5517	**0.3889**	0.4348	0.2233	**0.0637**	0.1734
	4	0.6986	**0.4409**	0.4948	0.4193	**0.0635**	0.2645	0.5517	**0.3814**	0.4347	0.2233	**0.0555**	0.1690
	5	0.6986	**0.4405**	0.4873	0.4193	**0.0610**	0.2636	0.5517	**0.3811**	0.4286	0.2233	**0.0514**	0.1697
Eye Color	1	0.8475	**0.4169**	0.4439	0.6137	**0.0368**	0.2340	0.6239	**0.3522**	0.3837	0.3435	**0.0428**	0.1678
	2	0.8475	**0.4098**	0.4415	0.6137	**0.0274**	0.2341	0.6239	**0.3478**	0.3823	0.3435	**0.0296**	0.1683
	3	0.8475	**0.4077**	0.4381	0.6137	**0.0248**	0.2341	0.6239	**0.3475**	0.3809	0.3435	**0.0250**	0.1680
	4	0.8475	**0.4033**	0.4351	0.6137	**0.0226**	0.2340	0.6239	**0.3441**	0.3789	0.3435	**0.0231**	0.1681
	5	0.8475	**0.4034**	0.4313	0.6137	**0.0230**	0.2341	0.6239	**0.3443**	0.3767	0.3435	**0.0222**	0.1677
